# Conversion between 100-million-year-old duplicated genes contributes to rice subspecies divergence

**DOI:** 10.1186/s12864-021-07776-y

**Published:** 2021-06-19

**Authors:** Chendan Wei, Zhenyi Wang, Jianyu Wang, Jia Teng, Shaoqi Shen, Qimeng Xiao, Shoutong Bao, Yishan Feng, Yan Zhang, Yuxian Li, Sangrong Sun, Yuanshuai Yue, Chunyang Wu, Yanli Wang, Tianning Zhou, Wenbo Xu, Jigao Yu, Li Wang, Jinpeng Wang

**Affiliations:** 1 School of Life Sciences, and Center for Genomics and Computational Biology, North China University of Science and Technology, Tangshan, 063000 Hebei China; 2 University of Chinese Academy of Sciences, Beijing, 100049 China; 3 State Key Laboratory of Systematic and Evolutionary Botany, Institute of Botany, Chinese Academy of Science, Beijing, 100093 China

**Keywords:** Rice, Polyploidization, Whole-genome duplication, Duplicated genes, Ongoing gene conversion

## Abstract

**Background:**

Duplicated gene pairs produced by ancient polyploidy maintain high sequence similarity over a long period of time and may result from illegitimate recombination between homeologous chromosomes. The genomes of Asian cultivated rice *Oryza sativa ssp. indica* (XI) and *Oryza sativa ssp. japonica* (GJ) have recently been updated, providing new opportunities for investigating ongoing gene conversion events and their impact on genome evolution.

**Results:**

Using comparative genomics and phylogenetic analyses, we evaluated gene conversion rates between duplicated genes produced by polyploidization 100 million years ago (mya) in GJ and XI. At least 5.19–5.77% of genes duplicated across the three rice genomes were affected by whole-gene conversion after the divergence of GJ and XI at ~ 0.4 mya, with more (7.77–9.53%) showing conversion of only portions of genes. Independently converted duplicates surviving in the genomes of different subspecies often use the same donor genes. The ongoing gene conversion frequency was higher near chromosome termini, with a single pair of homoeologous chromosomes, 11 and 12, in each rice genome being most affected. Notably, ongoing gene conversion has maintained similarity between very ancient duplicates, provided opportunities for further gene conversion, and accelerated rice divergence. Chromosome rearrangements after polyploidization are associated with ongoing gene conversion events, and they directly restrict recombination and inhibit duplicated gene conversion between homeologous regions. Furthermore, we found that the converted genes tended to have more similar expression patterns than nonconverted duplicates. Gene conversion affects biological functions associated with multiple genes, such as catalytic activity, implying opportunities for interaction among members of large gene families, such as NBS-LRR disease-resistance genes, contributing to the occurrence of the gene conversion.

**Conclusion:**

Duplicated genes in rice subspecies generated by grass polyploidization ~ 100 mya remain affected by gene conversion at high frequency, with important implications for the divergence of rice subspecies.

**Supplementary Information:**

The online version contains supplementary material available at 10.1186/s12864-021-07776-y.

## Background

Rice is the largest food crop in the world. There are two distinct types of domesticated rice, Asian rice (*Oryza sativa* L*.*) and African rice (*Oryza glaberrima* L.), each with unique histories of domestication [1]. Asian rice is planted worldwide, feeding half of the world’s population as a staple food and providing more than 20% of the energy for human survival [2–4].*Oryza sativa ssp. indica* (XI) and *Oryza sativa ssp. japonica* (GJ) are the two major subspecies of rice; they diverged ~ 0.4 million years ago (mya) [5]. The first whole-genome draft sequence of the GJ cultivar ‘Nipponbare’, which is representative of the subspecies, was obtained in 2002 [6], and genome sequencing and annotation have been continuously improved [7]. The whole-genome sequence of XI (93–11) has also been published [8], and high-quality genome sequences of representative varieties Zhenshan 97 (XI-ZS97) and Minghui 63 (XI-MH63) have been made available [9]. These two main varieties of XI (XI-ZS97 and XI-MH63) are the parents of an excellent Chinese hybrid. XI accounts for more than 70% of global rice production and possesses much higher genetic diversity than GJ [10], as highlighted by a recent analysis of 3010 diverse Asian cultivated rice genomes and 1275 rice varieties with resequenced genomes [4, 11].

Polyploidy or whole-genome duplication (WGD) is the doubling of an entire set of chromosomes in cells and is prevalent throughout the plant and animal kingdoms [12]. The impact of polyploidization on plant functional evolution is extremely profound, facilitating rapid expansion and divergence of species [13–16]. A large number of duplicated genes generated by polyploidization are distributed on homeologous chromosomes in extant species, which leads to genome instability. Homoeologous recombination as an evolutionary event of instability can lead to gene loss [17, 18], de novo functionalization of genes, subfunctionalization [19], or rearrangement of genomic DNA [20–23], providing material for plant evolution. In modern rice, at least five WGD events occurred during the evolution. The oldest WGD event (ζ) is shared by seed plants (~ 310 mya) and another WGD event (ε) that occurred prior to the appearance of all extant angiosperms (~ 235 mya) [13]. Two relatively recent WGD events occurred after the formation of monocotyledons: one (τ) shared by most monocotyledons at ~ 130 mya and another (σ) shared by Poales at ~ 115–120 mya [17, 24, 25]. The most recent WGD event (ρ) was originally thought to have occurred before the divergence of major grasses (~ 70 mya) [17, 20]; however, the latest fossil evidence advances this ρ event to ~ 100 mya and identifies the most common duplicated genes related to these WGD events [25].

Homologous recombination provides a major source for genetic innovation [26]. In plants, meiotic and mitotic recombination result in the reciprocal or symmetric exchange of DNA sequence information between homologous chromosomes [27]. In addition to homologous recombination, highly similar sequences undergo frequent recombination between homeologous chromosomes, which is called illegitimate recombination [28]. One result of this recombination is gene conversion, where one gene (or DNA segment) replaces another gene (or DNA segment) on a homeologous chromosome or chromosomal region. Gene conversion between duplicated genes produced by WGD has been identified in the genomes of *Poaceae*, *Arachis hypogaea*, *Gossypium*, *Brassica campestris*, and *Brassica oleracea* [18, 28–32]. In addition, gene conversion is frequent and ongoing between homologous chromosomes, such as homeologous chromosomes 11 and 12 produced from the duplication common to grasses (ρ event) in the modern rice genome [26, 28, 33, 34].

Recombination is a mutagenic factor, and mutations lay the foundation for natural selection. The main role of gene conversion is to maintain the homology or similarity of duplicated sequences. A comparison between rice and sorghum genomes clearly suggests that gene conversion promotes species divergence [28] because recombination accelerates mutation, with gene conversion playing an important role [35]. Gene conversion of functional sequences and new mutations produced by related homeologous recombination may affect gene function. For example, studies have revealed that sequences encoding functional domains are converted more frequently than those encoding nonfunctional domains [36]. Gene conversion and DNA duplication may facilitate functional innovation through gene extension and mutations in structural domains of disease-resistance genes [37]. Gene conversion between chromosomes 11 and 12 of rice has been accompanied by subfunctionalization or purifying selection of genes related to spikelet abortion [38], lipid transfer [39, 40], recessive yellowing control [41], cyclic C2-type proteins [42], and the zinc-inducible promoter family [43].

Our knowledge of gene conversion between paralogous genes in the two rice subspecies is based on outdated genomic data (ver. 4) [36]. Here, we used the latest genomic data and recent approaches for resolving genomic homology [22] to identify paralogous genes generated by grasses comment WGD event (ρ) in three rice genomes representing the two major subspecies. Then, comparative and phylogenetic genomics was combined to establish an improved method for inferring gene conversion between the 100 mya duplicated genes. We evaluated the ratio, level, and pattern of gene conversion in three rice genomes and explored the effects of conversion on the genome evolutionary rate, gene expression and functional innovation, chromosome structure, and genome stability.

## Methods

### Materials

Genomic sequence data for XI-MH63, XI-ZS97, *Sorghum bicolor* [44] and *Brachypodium distachyon* [45] were obtained from the GenBank database (https://www.ncbi.nlm.nih.gov/). The GJ (Nipponbare), *Setaria italica* [46] and *Setaria viridis* [47] genomes were downloaded from Gramene (http://www.gramene.org/), and the *Arabidopsis thaliana* genome was downlanded from TAIR (https://www.arabidopsis.org/).

### Detection of colinear genes and construction of homologous gene quartets

To identify collinear genes within the genome and between genomes, first, BLASTP [48] was used to search for intragenomic and intergenomic homology of protein sequences, and an E-value <1e-5 and score > 100 were strictly set to exclude more-diverged homologous genes and remove shorter matched gene pairs. Then, ColinearScan [20] was employed to analyse collinear regions based on gene homology predictions, and the significance of collinearity was tested. The key parameter, the maximum gap, was set to 50 intervening genes, and genes in large gene families with 30 or more copies in a genome were removed from the inferring collinearity, as adopted in our previous genomics research [22, 49, 50]. Based on our previous research in rice, 100 million-year-old paralogous blocks produced in the grass common ancestor [25] were identified in three rice genomes. In addition, to obtain more complete homology information within genomes, further bidirectional best BLASTP homology searches were performed on the three genomes. Then, we performed a two-way comparison through a homologous dot plot to help distinguish orthologous and paralogous genomes. Additionally, we obtained paralogues and orthologues within each genome and between genomes.

**Fig. 1:**
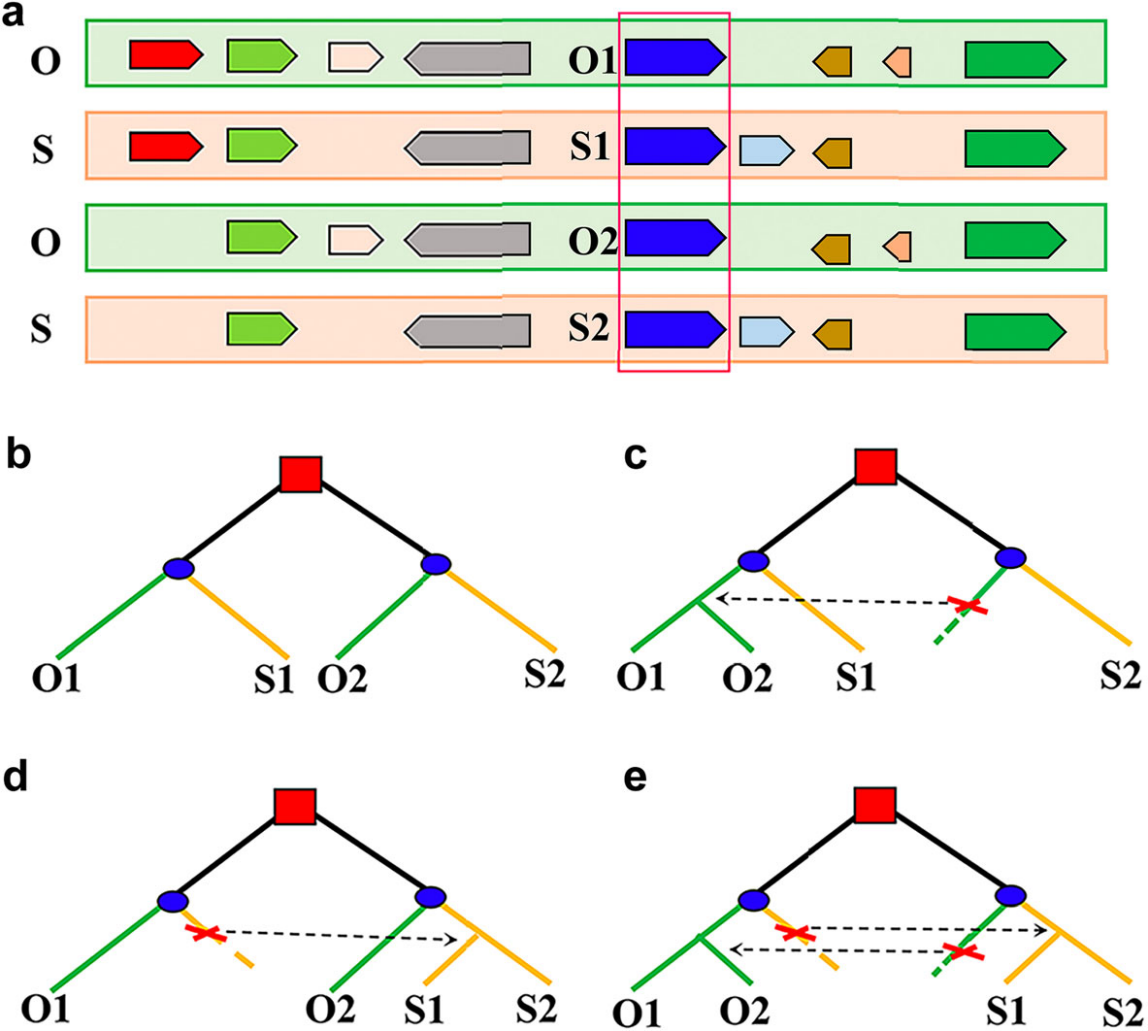
Gene conversion events of homologous gene quartets and changes in phylogenetic tree topology. **a** Colinear chromosomal segments from two genomes (O and S), represented by rectangles of different colours. Arrows show genes, and homologous genes are indicated by the same colours. Homologous gene quartets are formed by paralogous genes O1 and O2 in one genome and their respective orthologues S1 and S2 in the other genome. (b-e) Squares symbolize a WGD event in the common ancestral genome; circles symbolize species divergence. **b** The expected phylogenetic relationship of the homologous genes if no conversion occurs. **c** O2 (an acceptor) is converted by O1 (a donor). **d** S1 is converted by S2. **e** Both of the above conversions occur

To infer the potential gene conversion between duplicated genes produced by WGD, we defined ‘homologous gene quartets’. Assuming that the two rice subspecies, O and S, both retain a pair of duplicated chromosomal segments generated in their common ancestor through WGD, then the paralogous genes O1 and O2 and their respective orthologous genes S1 and S2 comprise a homologous gene quartet (Fig. 1a).

### Ks and Ka calculation

The synonymous nucleotide substitution rate (*Ks*) and nonsynonymous nucleotide substitution rate (*Ka*) values were estimated by using the Nei-Gojobori approach [51] implemented in PAML v.4.9 h [52]. Considering that nucleotide substitutions occur frequently at some sites in a sequence, the multiple substitutions may occur at these sites, the Jukes-Cantor (JC) model was employed to correct the *Ks* and *Ka* values, and they are denoted as Ps and Pa [28, 53].

### Inference of gene conversion

To infer possible gene conversion between duplicated genes, the multiple sequence alignment software ClustalW [54] was employed to compare the identity between homologous gene sequences in quartets. Highly divergent quartets were removed to eliminate potential problems created by inferring gene conversion from unreliable sequences. Quartets showing gaps in the pairwise alignments exceeding 50% of the alignment length or with amino acid identity between homologous sequences of less than 40% were removed.

*Whole-gene conversion* (WCV) inference: Since paralogues were produced prior to subspecies divergence, we anticipate that the orthologues between two subspecies should be more similar than the paralogues in each subspecies. If the paralogues were more similar to one another than to their respective orthologues across subspecies, we inferred that gene conversion occurred after species divergence. For this comparison, phylogenetic analyses of homologous gene quartets were performed to infer potential whole gene conversion between paralogues according to the gene topology changes (Fig. 1b-e). To measure the similarity of the homologous genes in each quartet, we characterized the *Ks* values and ratios of amino acid locus identity between paralogues and orthologues. First, the *Ks* values between paralogous and orthologous gene pairs were used to infer possible whole gene conversion (called WCV-I). To assess the confidence level of the inferred conversions, bootstrap tests were performed on each gene tree with 1000 repetitive samplings to produce a bootstrap frequency [28, 33]. Second, the sequences in quartets were compared site by site and then used to calculate the ratios of amino acid locus identity between paralogous and orthologous gene pairs. The ratios were used to infer unexpected changes in gene tree topology in quartets, depending on whether the paralogues were more similar to each other than the orthologues [28]. This is a strict criterion used for the detection of whole gene conversion (called WCV-II), as paralogues were produced at least 100 mya, whereas orthologues have diverged more recently. The similarity between sequences representing different rice subspecies is often very high due to the relatively close genetic relationship between these three rice genomes, as in our previous study of the conversion between hexaploid wheat subgenomes [32].

*Partial-gene conversion* (PCV) inference: Quartets were used to identify possible gene conversion among partial gene sequences that may occur after species divergence. A combination of dynamic planning and phylogenetic analysis was used to document the differences between two aligned bases from paralogues within genomes and orthologues between genomes, as previously reported [28]. The main steps for inferring PCV include: 1) defining arrays to reflect the difference or distance between the homologues, and 2) averaging the distance arrays of the orthologous gene pairs and comparing the averaged distance between paralogues and orthologues, as the paralogues should be more distant if no PCV is involved. 3) Dynamic programming was used to reveal high-scoring segmental sequences and infer the extension of paralogues. Then, partially affected regions ≥10 nucleotides in length were identified. 4) A bootstrap test was used to assess the identification of high-scoring segments with shorter lengths and smaller scores. 5) After masking some of the larger segments, a recursive procedure revealed shorter high-scoring fragments, which helped to reveal genes affected by multiple conversion events.

The scripts of gene conversions inference have deposited in Github (https://github.com/weichendan312/gene-conversion).

### Statistical analysis of the distribution of converted duplicates

To analyse the correlation between gene conversions and their physical location on the chromosome, we investigated converted and nonconverted duplicates relative to their proximity to chromosomal termini. On each chromosome arm, duplicated genes were divided into 500 kb bins ordered from the chromosome termini to the centromere, and the number of converted and nonconverted genes in each bin was summed. Then, the conversion rates in each bin were calculated by dividing the converted gene number by the nonconverted gene number. The first selected bins on each arm (outermost region of each arm) were compared to all other bins on the chromosome. The fold increase in conversion rates was calculated by dividing the mean in the first selected bin by the mean of all other bins. A 1,000,000 round permutation test was performed by randomly exchanging the bin sums of conversion rates and calculating the fold increase for each permutation. This type of statistical analysis approach has previously been used to investigate the unbalanced distribution of human variants on chromosomes [55].

### Conversion and gene ontology analysis

To obtain a functional overview of the duplicated genes, InterProScan 5 [56] was used to determine the GO classification of each gene. All records are derived from the literature-based annotations and domain-based electronic annotations. GO annotation results of the converted and nonconverted gene sets were compared and plotted using the online visualization tool WEGO [57] to visualize the distribution of functional genes and trends. The significance of the difference in the number of functional duplicates between those with and without conversion genes was tested by Pearson’s chi-square test.

### Identification of disease-resistance genes

HMMscan [58] was employed to identify NBS-LRR domain gene set A in the GJ, XI-MH63, and XI-ZS97 genomes, and the genes with the NB-ARC domain (PF00931) were also searched [58] in the *Arabidopsis thaliana* genome. The key parameter E-value was set to 1e-10. Then, using the NBS-LRR genes of *Arabidopsis thaliana*, BLASTP was used to compare these sequences with the GJ, XI-MH63, and XI-ZS97 genomes, and the genes with E-values <1e-10 were designated NBS-LRR gene set B. Ultimately, genes present in both gene sets A and B were identified as NBS-LRR genes in the three rice genomes.

### Differential expression of gene conversions

Raw RNA-seq reads of rice subspecies were downloaded from NCBI. We downloaded the panicle (SRR13528769) and seedling (SRR13528768) for GJ, the leaf (SRR10751892) and panicle (SRR10751894) for XI-MH63, and the leaf (SRR10751907) and root (SRR10751904) for XI-ZS97. Raw RNA-Seq reads were preprocessed using Trimmomatic [59] to remove adaptor sequences and low-quality reads with the parameters ‘PE -phred33 ILLUMINACLIP: Trimmomatic-0.36/adapters/TruSeq3-PE.fa: 2:30:10 LEADING: 20 TRAILING: 20 SLIDINGWINDOW: 4:20 MINLEN: 50’. The clean reads were then mapped to the three rice genomes using HISAT2 [60] with default parameters. The expression abundance values were calculated using StringTie [61] with options ‘-e -A’. To compare the expression patterns of converted and nonconverted duplicates, we removed genes with no expression, and then the log2 was taken for the standardization of gene expression values.

## Results

### Intra/intergenomic homologous genes

Through intragenomic and intergenomic collinearity analysis, we identified collinear genes in the GJ, XI-MH63, and XI-ZS97 genomes. For blocks containing more than four collinear gene pairs, there were more homologous gene pairs in GJ (3314 pairs) than in the two XI genomes, which included 2629 and 2889 homologous gene pairs, respectively (Additional file 1: Table S1). Comparisons of the homologous blocks with more than four, 10, 20, and 50 colinear gene pairs in the three genomes showed that the XI genomes had much shorter duplicated blocks than the GJ genome. In the GJ, XI-MH63, and XI-ZS97 genomes, 10, 10, and 9 blocks had more than 50 colinear gene pairs which contained 1373, 1842, and 1894 colinear genes, respectively. Notably, we also found that the average colinear gene pairs per block were shorter in the two XI genomes than in the GJ genome. This indicated that the two varieties of XI have poor collinearity, which may be due to more genomic rearrangements in their genomes [9]. Using these inferred collinear genes of the three rice genomes, we identified duplicated genes produced by the WGD common to the grasses at ~ 100 mya, according to the sequence similarity of collinear gene pairs and our previous identification of WGD event-related homologous chromosome regions [62]. Since some potential paralogues might have been removed from the paralogous regions in the collinearity analysis, we also used a bidirectional best BLAST search of three genomes to identify the matching homologous gene pairs that were added to the paralogous regions. Finally, 3256, 2502, and 2816 duplicated gene pairs were identified in the GJ, XI-MH63, and XI-ZS97 genomes, respectively (Fig. 1a-c).

We also inferred collinear genes between any two rice genomes (Additional file 2: Table S2). According to the sequence similarity of collinear gene pairs and our previous identification of homologous chromosome regions generated by recent rice subspecies divergence, we identified orthologous genes between any two rice genomes. A total of 19,089 orthologous gene pairs persisted in 103 blocks between GJ and XI-MH63. The two XI genomes showed better orthologous collinearity, with 25,262 orthologous gene pairs persisting in 146 blocks. We also added the bidirectional best BLAST matching homologous gene pairs in orthologous regions. There were 23,719 orthologous gene pairs between GJ and XI-MH63 and 23,056 orthologous gene pairs between GJ and XI-ZS97. Since XI-MH63 and XI-ZS97 are more closely related, we identified more orthologous gene pairs (35,049) between the genomes of XI varieties.

### Homologous gene quartets

To detect possible gene conversion between homologous genes produced by WGD, we used homology and collinearity information to construct homologous gene quartets between any two rice genomes (Fig. 1a). In a quartet, sequence similarity between orthologues is more similar than that between paralogues if there is no gene conversion (or nonreciprocal recombination) between the duplicates after subspecies divergence (Fig. 1b). However, if gene conversion occurs between duplicated genes, we might find that the gene tree topology has a different structure than expected (Fig. 1c-e). Changes in the topological structure of the gene tree can be determined from the similarity of homologous sequences in quartets (see the Methods for details).

**Fig. 2:**
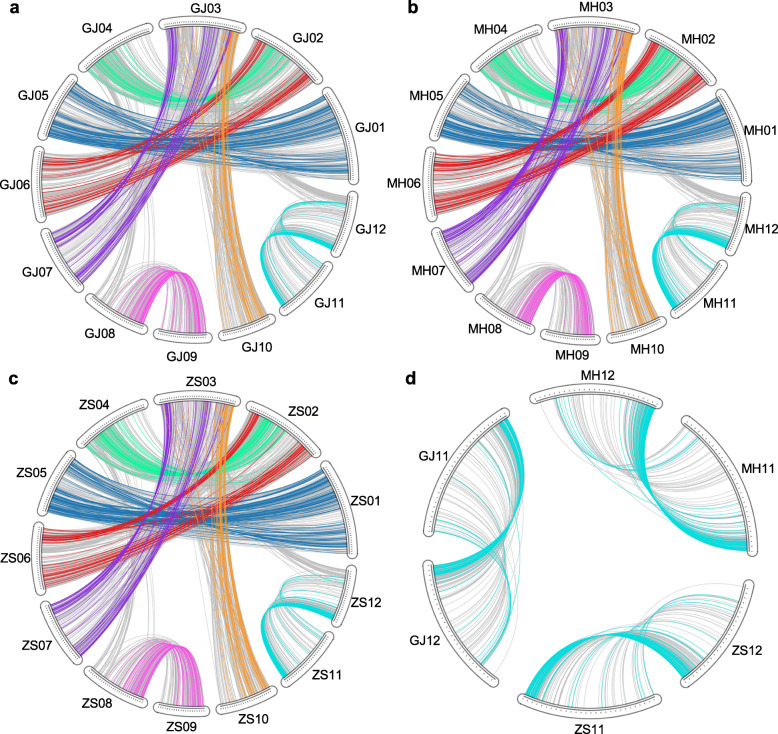
Genome duplications and conversion patterns in three rice subspecies genomes. Lines show duplicated gene pairs between chromosomes across three genomes. Coloured lines indicate converted gene pairs; grey lines indicate duplicated gene pairs. **a** Gene duplication and gene conversion in GJ. **b** Gene duplication and gene conversion in XI-MH63. **c** Gene duplication and gene conversion in XI-ZS97. **d** Gene duplication and gene conversion on chromosomes 11 and 12 of GJ, XI-MH63, and XI-ZS97

Based on collinearity information of intragenomic and intergenomic comparisons, we identified 2788 quartets between GJ and XI-MH63 and 2879 quartets between GJ and XI-ZS97. Although XI-MH63 and XI-ZS97 are varieties of the same subspecies, relatively few quartets (2566) were identified between them, probably due to differences in gene loss after the three genomes diverged. By comparing the three genomes, we inferred a possible ancestral gene content before divergence of 19,104 genes. The rates of gene loss or translocation were 6.13, 13.31, and 7.89% in GJ, XI-MH63, and XI-ZS97, respectively. Finally, we identified 3332, 3322, and 3254 homologous genes from quartets in GJ, XI-MH63, and XI-ZS97, respectively. These homologous genes were mainly conserved in 82, 85, and 93 blocks, and they were unevenly distributed across the 12 chromosomes in the three genomes (Fig. 2).

### Gene conversion and occurrence patterns

We removed highly divergent sequences to reduce the possibility of inferring gene conversion events from unreliable sequences (see Methods for details). After this, 2788 gene quartets were identified between GJ and XI-MH63, 2879 quartets were identified between GJ and XI-ZS97, and 2566 quartets were identified between XI-ZS97 and XI-MH63 (Additional file 2: Table S2). We used two methods to infer gene tree topology, one based on the synonymous nucleotide substitution rate (*Ks*) as a similarity measure and the other based on the amino acid identity ratio, which we called whole-gene conversion type I and type II (WCV-I and WCV-II), respectively. We used a combination of dynamic planning and phylogenetic analysis to infer possible partial-gene conversion (PCV) events (Additional file 3: Table S3). Since paralogous gene pairs may be identified in different quartets, we merged the paralogous gene pairs affected by gene conversion in each genome. This gave us both potential gene conversion events between paralogues in each genome after the divergence of rice.

**Fig. 3:**
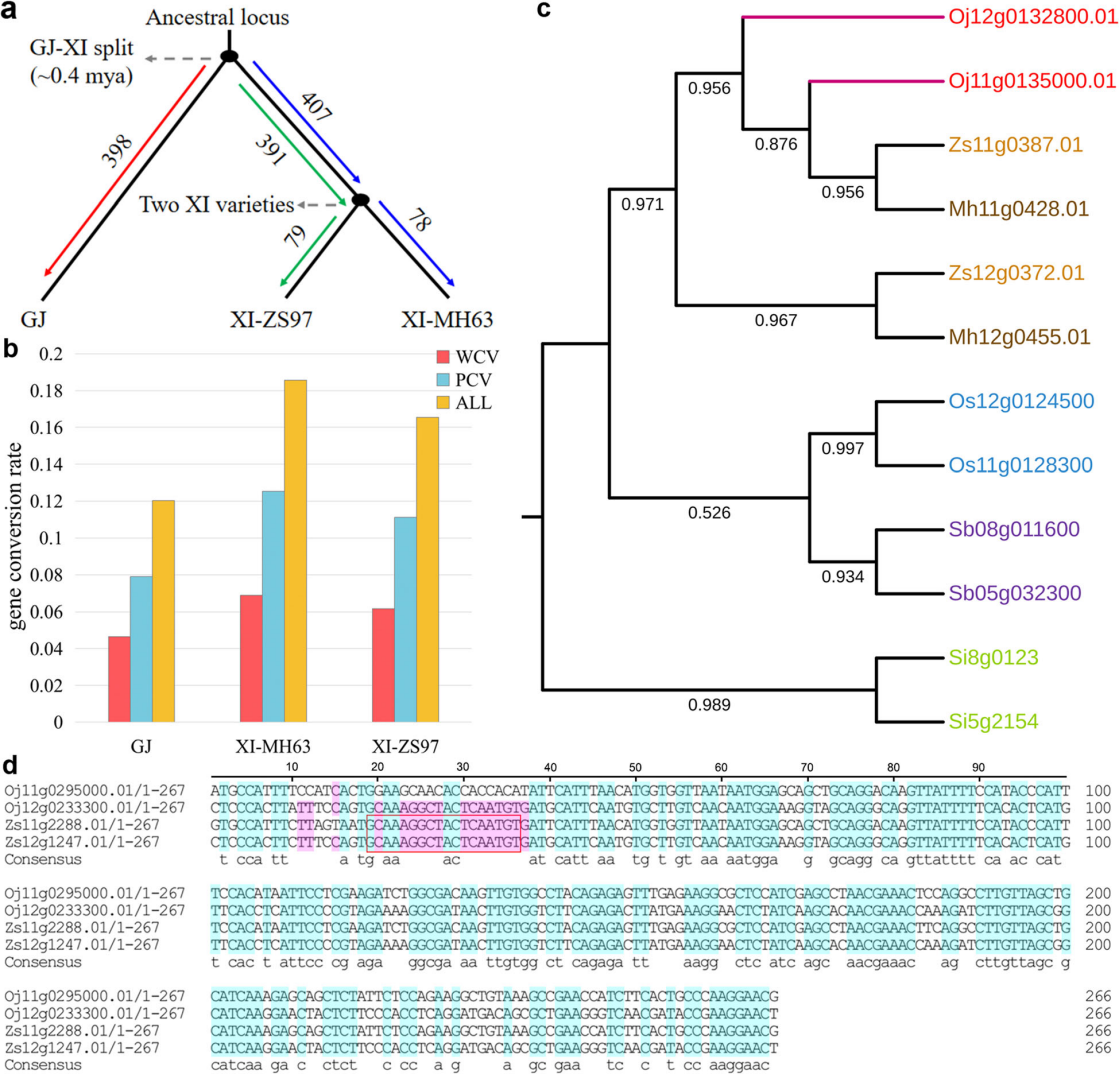
Evolution of gene conversion. **a** Gene conversion in species divergence events. The numbers on different coloured lines indicate the number of gene conversions in different species at different times. **b** The rates of WCV and PCV in the three genomes. In the histogram, orange represents the number of all gene conversions, blue represents the number of PCVs, and red represents the number of WCVs. **c** Evolutionary trees of genes in which gene conversion occurred. The gene *Oj12g0106200.01* was wholly converted by its paralogous gene *Oj11g0106900.01* in GJ. **d** Multiple sequence alignment of one selected quartet. The blue highlighted areas indicate 100% similarity, and the red highlighted areas indicate 75% similarity. The 19–36 bp nucleotide sites of *Zs11g2288.01* and *Zs12g1247.01* underwent gene conversion, and the donor was *Zs12g1247.01*. The sequence in the red rectangle indicates partial gene conversion

In GJ, 398 pairs (~ 12%) of paralogues were converted after divergence with XI (Fig. 3a). Of these, 179 pairs (5.37%) had undergone WCV: 11 pairs were inferred by WCV-I, and 168 pairs were inferred by WCV-II. Another 259 pairs (7.77%) had undergone PCV, which occurred at a higher rate than WCV. In XI-MH63, 466 pairs (~ 14%) of paralogues had been converted, of which 182 pairs (5.48%) had undergone WCV: 8 pairs were inferred by WCV-I and 174 pairs were inferred by WCV-II. Another 312 pairs (9.39%) had undergone PCV, which was remarkably higher than WCV. Similar to XI-MH63, 468 pairs (~ 14%) of paralogues had been converted in XI-ZS97: 185 pairs (5.69%) had undergone WCV, comprising 8 pairs inferred by WCV-I and 177 pairs inferred by WCV-II. Another 310 pairs (9.53%) had undergone PCV, which was also higher than WCV (Table 1). A comparison of the converted paralogues between GJ and XI revealed that the gene conversion rates were 0.19 and 0.17 in the two XI genomes, respectively, which were higher than that in GJ (0.12) (Fig. 3b). Two examples further illustrate the conversion patterns of paralogues: first, we detected the gene *Oj12g0132800.01* wholly converted by its paralogous gene *Oj11g0135000.01* in GJ, which was also supported by gene topological structure changes in the gene trees (Fig. 3c). Second, we detected one paitial converted paralogous gene pairs *Zs11g2288.01* and *Zs12g1247.01* in XI-ZS97, and one DNA segment from 19 to 36 bp of *Zs11g2288.01* was converted by *Zs12g1247.01* (Fig. 3d).

**Table 1:** Converted paralogues in GJ and two XI (XI-MH63 and XI-ZS97) genomes

	GJ	XI-MH63			XI-ZS97		
		Period A^a^	Period B^b^	Total	Period A^a^	Period B^b^	Total
Paralogues	3332	3322			3254		
WCV-I	11 (2.76%)	7 (1.72%)	1 (1.28%)	8 (1.72%)	7 (1.79%)	1 (1.27%)	8 (1.71%)
WCV-II	168 (42.21%)	173 (42.51%)	1 (1.28%)	174 (37.34%)	175 (44.76%)	2 (2.53%)	177 (37.82%)
PCV	259 (65.08%)	250 (61.43%)	77 (98.72%)	312 (66.95%)	251 (61.22%)	77 (97.47%)	310 (66.24%)
Chr. 11 and 12 CV^c^	64 (16.08%)	63 (15.48%)	17 (21.79%)	76 (16.31%)	57 (14.58%)	8 (10.13%)	64 (13.68%)
All converted	398	407	78	466	391	79	468
Conversion rate	0.119	0.123	0.023	0.140	0.120	0.024	0.144

^a^Conversion events occurred after the GJ-XI divergence, and before the formation of the two XI varieties

^b^Conversion events occurred after the formation of the XI varieties (XI-MH63 and XI-ZS97)

^c^Converted paralogues distributed on chromosomes 11 and 12 in the three rice genomes

### High-frequency on-going gene conversion

Duplicated gene pairs produced ~ 100 mya persist in the three rice genomes and are still being affected by recent gene conversion events. In GJ, we identified 398 pairs of paralogous genes that might have undergone gene conversion after the divergence of GJ from XI. The amino acid identity of four (1.01%) pairs of converted paralogous genes was > 99%, with *Ks* < 0.01. A relatively large number of duplicated genes were affected by gene conversion in the two XI variety genomes. In XI-MH63, we found 466 pairs of paralogous genes that might have undergone gene conversion after the divergence of GJ from XI. Six (1.29%) of these pairs of paralogous genes had > 99% amino acid identity between them and *Ks* < 0.01. Similarly, we also found a similar pattern in XI-ZS97. We identified small synonymous and nonsynonymous nucleotide substitutions and high sequence identity between duplicated gene pairs in which gene conversion had occurred, suggesting the possibility of having very recently been converted.

Another striking indication was that 407 and 391 pairs of paralogous genes were affected by gene conversion before the formation of XI-MH63 and XI-ZS97, respectively. Seventy-eight and 79 pairs of paralogous genes were converted after the formation of the two XI varieties, accounting for 16.7 and 16.6% of the total gene conversion, respectively (Fig. 3d). Many duplicated gene pairs in GJ, XI-MH63 and XI-ZS97 showed nearly 99% amino acid identity, and *Ks* was less than 0.1 (Additional file 4: Fig. S1; Additional file 5: Fig. S2). These pairs deviated far from the peak *Ks* location at ~ 0.65 for the duplicated genes produced by the WGD common to grasses [62]. These data suggest that gene conversion between duplicated gene pairs is ongoing and occurs at high frequencies in rice subspecies.

### Donor genes are biased towards donating

**Fig. 4:**
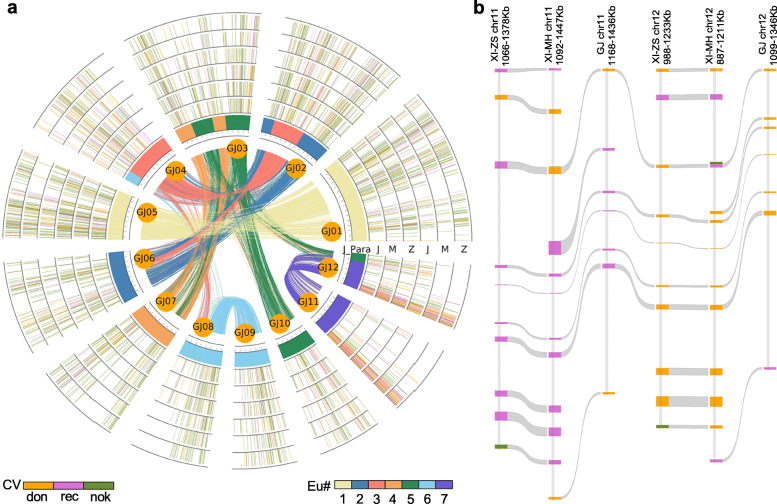
Distribution of donors and receptors in the genome where gene conversion occurs. **a** Homologous distribution of donors and acceptors on chromosomes undergoing gene conversion. Curved lines within the inner circle are formed by 12 chromosomes of GJ colour coded to the seven ancestral chromosomes before the WGD event common to grasses [62]. Intra-loop curves show duplicated gene pairs in GJ. The three inner circles show the relationships of orthologous gene distribution between the three genomes in which gene conversion has occurred. The three outer circles show the distribution between the three genomes undergoing gene conversion, and the three inner circles show paralogous homologues. Different colours indicate donor (orange) or acceptor (pink) loci, as well as some uncertain loci (green). Among them, J_para represents the paralogous genes in GJ, J represents GJ, M represents XI-MH63, and Z represents XI-ZS97. **b** Local gene conversion and the distribution of donor and acceptor loci. Pink swatches represent donor loci, orange swatches represent acceptor loci, and green swatches represent those loci where donor or acceptor status is uncertain

Gene conversion involves a donor locus and an acceptor locus. Donors and acceptors can be identified by comparing topological changes in the phylogenetic trees of homologous gene quartets since the paralogue of the donor should be more similar than its orthologue in another subspecies (Fig. 1c-d). We inferred possible donor genes for 196, 215, and 200 converted duplicated genes in GJ*,* XI-MH63, and XI-ZS97, respectively,. A total of 1520 duplicated genes were converted across the three genomes, with 1378 (90.66%) of these converted in at least two genomes, and they are shown in a circle (Fig. 4a). Interestingly, 88.98% (113/127) of the genes were taken as donors in at least two genomes, and 66.93% (85/127) of the genes had the same donor in all three genomes (Additional file 6: Table S4). This suggested that the donor genes in converted paralogues are often taken as donor loci in different genomes. For example, there were 13 duplicated genes in the region of ~ 1.0 Mb near the telomere on chromosomes 11 and 12 across the three rice genomes. A total of 92.31% (12/13) of the duplicated genes were affected by gene conversion in at least two genomes. A total of 76.92% (10/13) of the duplicated genes were taken as donors, and 70% (7/10) of the duplicated genes acted as donors in different genomes (Fig. 4b).

### Unblanced distribution of converted duplicates

Gene conversion was unevenly distributed across the different homologous chromosomal regions, and all three genomes were most affected by gene conversion between duplicated genes on chromosomes 11 and 12. The gene conversion rates were 18.88, 21.78, and 18.71% on chromosomes 11 and 12 of GJ, XI-MH63, and XI-ZS97, respectively (Additional file 7: Table S5). In addition, the duplicated genes located at 2 Mb at the termini of chromosomes 11 and 12 were likely most affected by gene conversion, and the gene conversion rates were approximately 70% in the three rice genomes (Fig. 2d).

The physical location of paralogues on chromosomes may correlate with their chance of being converted. We observed that duplicated gene pairs were often distributed in distal regions on chromosomes, and the paralogue bias was affected by conversion near the ends of chromosomes (Fig. 2a-d). We estimated a 1.4-fold increase in the conversion rate within the subtelomeric region of 0–2 Mb to chromosome termini in GJ (*P*-value = 4.00 × 10^− 05^, permutation). In XI-MH63 and XI-ZS97, there was a 1.3- and 1.2-fold increase in the conversion rate within the subtelomeric region of 0–6 Mb to chromosome termini, respectively (*P*-value = 3.70 × 10^− 05^ and *P*-value = 1 × 10^− 04^, permutation) (Table 2). This indicates that duplicated genes within regions are the chromosomal termini more frequently affected by gene conversion in rice or subspecies genomes.

**Table 2:** Relationship between gene physical location and gene conversion

Distance to telomere	< 2 Mb	2–4 Mb	4–6 Mb	6–8 Mb	8–10 Mb	> 10 Mb	All
GJ							
All converted	192	156	134	80	61	142	765
Paralogous genes	1270	1128	1092	781	595	1460	6326
Mean converted rate	15.13%	13.68%	12.28%	10.29%	9.94%	8.92%	12.09%
XI-MH63							
All converted	217	192	151	105	69	175	909
Paralogous genes	1076	909	776	652	481	1002	4896
Mean converted rate	20.35%	21.26%	19.65%	17.34%	15.95%	16.55%	18.57%
XI-ZS97							
All converted	214	185	154	103	80	176	912
Paralogous genes	1234	1041	862	704	538	1107	5486
Mean converted rate	17.35%	17.76%	17.55%	14.65%	14.88%	15.43%	16.62%

### Effect of chromosome rearrangement on gene conversion

**Fig. 5:**
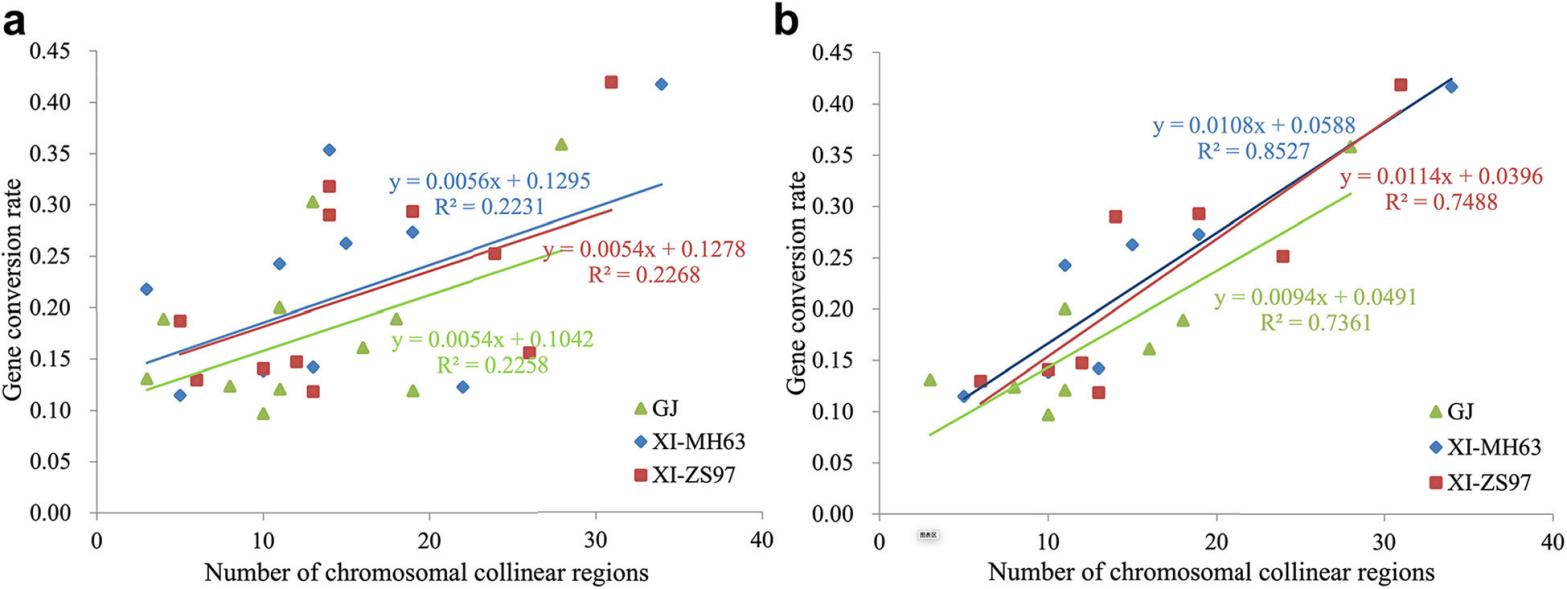
Relationship between block number and gene conversion rate on each chromosome. **a** Relationship between block number on 12 chromosomes and the gene conversion rate on the corresponding chromosomes of GJ, XI-MH63, and XI-ZS97. **b** Relationship between block number on 8 chromosomes and the gene conversion rate on the corresponding chromosomes after removing the four special chromosomes (homologous chromosome pair 1–5 and homologous chromosome pair 11–12)

Chromosome rearrangement is a random evolution event, and the block number in the genome can reflect the degree of chromosome rearrangement after polyploidization. We found that block number and gene conversion rate showed a weak positive correlation in XI-MH63 (*R*^*2*^ = 0.22, *P*-value = 0.12), XI-ZS97 (*R*^*2*^ = 0.23, *P*-value = 0.11), and GJ (*R*^*2*^ = 0.11, *P*-value = 0.29) (Additional file 8: Table S6; Fig. 5a). If two special homeologous chromosome pairs (1–5 and 11–12) were removed, there was a significant positive correlation between block number and gene conversion rate in XI-MH63 (*R*^*2*^ = 0.85, *P*-value < 0.01), XI-ZS97 (*R*^*2*^ = 0.75, *P*-value < 0.01) and GJ (*R*^*2*^ = 0.74, *P*-value < 0.01) (Fig. 5b).

However, the correlation does not imply a direct factor leading to gene conversion. For this reason, we further analysed the relationship between block length and the gene conversion rate on each chromosome (Additional file 9: Table S7). We found that longer blocks had a higher gene conversion rate (Additional file 10: Fig. S3). The average gene conversion rate for a total of 14 blocks with more than 100 paralogous gene pairs was 14.12% (349 pairs). The block with fewer than 20 paralogous gene pairs was block 219, with a gene conversion rate of 11.77% (178 pairs). These results suggest that chromosome rearrangements are associated with ongoing gene conversion events, which may have contributed directly to restricting recombination between homoeologous regions and further inhibiting duplicated genes affected by conversion.

### Gene conversion and evolution

Gene conversion homogenizes paralogous gene sequences. This makes the affected paralogues appear younger than expected based on sequence divergence with one another. The Pn and Ps between paralogues undergoing gene conversion were smaller than those between paralogues not affected by gene conversion (Table 3). For example, the average Pn = 0.198 and Ps = 0.456 for converted genes were significantly smaller than the average Pn = 0.253 and Ps = 0.509 for genes not converted in GJ (*P*-value = 3.71 × 10^− 26^, *P*-value = 8.00 × 10^− 20^, t-test). We could not determine whether converted genes evolved slowly based on the paralogues themselves, since pairwise distances between paralogues were converted. However, Pn and Ps were slightly larger between orthologous gene pairs affected by gene conversion than between orthologues not showing gene conversion. This suggests that the orthologues in which gene conversion has occurred have evolved faster than those not affected by gene conversion.

**Table 3:** Nucleotide substitution rates of quartets in rice subspecies

Paralogues	XI-MH63			XI-ZS97			GJ		
	Pn	Ps	Pn/Ps	Pn	Ps	Pn/Ps	Pn	Ps	Pn/Ps
Converted	0.180	0.444	0.405	0.179	0.448	0.400	0.198	0.456	0.434
Nonconverted	0.234	0.486	0.481	0.235	0.486	0.484	0.253	0.509	0.497
*P*-value	1.82 × 10^−18^	8.73 × 10^−9^	5.04 × 10^−9^	7.81 × 10^−20^	1.25 × 10^−7^	2.44 × 10^−11^	3.71 × 10^*−26*^	8.00 × 10^*−20*^	2.53 × 10^− 9^
Orthologues	XI-MH63 vs. GJ			XI-ZS97 vs. GJ			XI-MH63 vs. XI-ZS97		
	Pn	Ps	Pn/Ps	Pn	Ps	Pn/Ps	Pn	Ps	Pn/Ps
Converted	0.049	0.076	0.645	0.053	0.085	0.624	0.055	0.100	0.550
Nonconverted	0.023	0.036	0.639	0.025	0.038	0.658	0.014	0.022	0.636
*P*-value	5.61 × 10^−19^	9.63 × 10^−23^	5.73 × 10^−23^	1.76 × 10^−21^	1.23 × 10^−27^	4.02 × 10^−14^	6.25 × 10^−20^	2.58 × 10^−31^	2.48 × 10^−11^

We used Ps and Pn to investgate whether gene conversion was affected by evolutionary selection pressure. The ratio of Pn and Ps reflects the selection pressure between gene pairs during evolution. We compared the ratio of Pn and Ps between genes subjected to conversion and those with no conversion. The average ratio for XI-MH63 gene conversion was 0.41, and the average ratio of nonconverted paralogues was 0.48. This indicates that converted genes were subject to purifying selection (Table 3). The ratios for gene conversion in XI-ZS97 and GJ were also smaller than those for nonconverted genes. The selection pressure for gene conversion or no gene conversion did not change much. However, there was not much difference in the selection pressure between orthologous gene pairs of gene conversion and no gene conversion. No evidence suggests a change in the selection pressure of converted genes.

### Gene conversion and function

Some duplicated genes are preferentially affected by gene conversion. We performed Gene Ontology (GO) analyses of duplicated genes related to biological functions. The GO analysis revealed that some genes with specific functions preferred conversion, while gene conversion of some functional genes was avoided (Additional files 11, 12 and 13: Fig. S4-S6). We identified the GO terms of 765, 909, and 912 converted duplicated genes and 5262, 5224, and 5135 nonconverted duplicated genes in GJ, XI-MH63, and XI-ZS97, respectively (Additional file 14: Table S8). We found that the genes involved in functions associated with large numbers of genes were biased towards gene conversion in the three genomes, such as catalytic activity, binding, metabolic process, and cellular process, which contained the largest percentage of duplicated genes (Additional file 14: Table S8). These four secondary-level terms were significantly enriched at the level of molecular function and biological processes and accounted for approximately 30% of the corresponding duplicated genes. For example, the number of catalytic activity and metabolic process genes in the three genomes in which gene conversion occurred (31.4–37.7%) was significantly greater than that in which no gene conversion occurred (26.6–30.6%) (*P*-value < 0.001, Pearson’s chi-square test) (Table 4). Similarly, the number of binding genes and cellular process genes showed higher gene conversion (27.4–39.9%) than duplicated genes without gene conversion (24.6.6–38.4%), suggesting that they are likely to be converted. In contrast, some genes associated with functions encoded by few genes (protein-containing complex, transporter activity) might have avoided gene conversion (Additional file 14: Table S8).

**Table 4:** Comparisons of the top four functions involved in converted genes compared with the nonconverted genes in the GJ, XI-MH63, and XI-ZS97 genomes

GO level2	GJ		XI-MH63		XI-ZS97	
	cv vs. non-cv	*P*-value	cv vs. non-cv	*P*-value	cv vs. non-cv	*P*-value
Catalytic activity	31.4 vs. 25.5	0.001	32.5 vs. 26.4	< 0.001	33.6 vs. 26.1	< 0.001
Binding	38.8 vs. 35.5	0.083	39.9 vs. 37.7	0.199	39.6 vs. 38.1	0.412
Metabolic process	37.7 vs. 27.7	< 0.001	36.8 vs. 29.6	< 0.001	37.4 vs. 29.4	< 0.001
Cellular process	28.9 vs. 24.0	0.003	28.0 vs. 26.2	0.262	27.4 vs. 25.8	0.32

Percentage of converted genes (cv), percentage of nonconverted genes (non-cv)

### Conversion and NBS-LRR genes

Rice diseases caused by various pathogens are one of the most serious constraints in global rice production [63]. Disease resistance genes play a very important role in the evolution of plant genomes and are one of the indispensable families of genes involved in the survival of plants under natural selection [64, 65]. We identified a total of 1697 NBS-LRR (nucleotide binding site-leucine rich repeat) resistance genes in the three genomes (Additional file 15: Table S9). Among these genes, we identified 462 NBS-LRRs in GJ, which was less than those identified in XI-MH63 (644) and XI-ZS97 (591). The NBS-LRR genes were unevenly clustered on the chromosomes of the three genomes. The NBS-LRR gene content on chromosome 11 was the highest, as confirmed in previous studies [3, 66]. Here, we found 113 (24.46%), 126 (21.32%), and 181 (28.11%) NBS-LRR genes on chromosome 11 of GJ, XI-MH63, and XI-ZS97, respectively. There were more NBS-LRR genes on chromosome 11 than on the other chromosomes (3.68–10.66%).

**Fig. 6:**
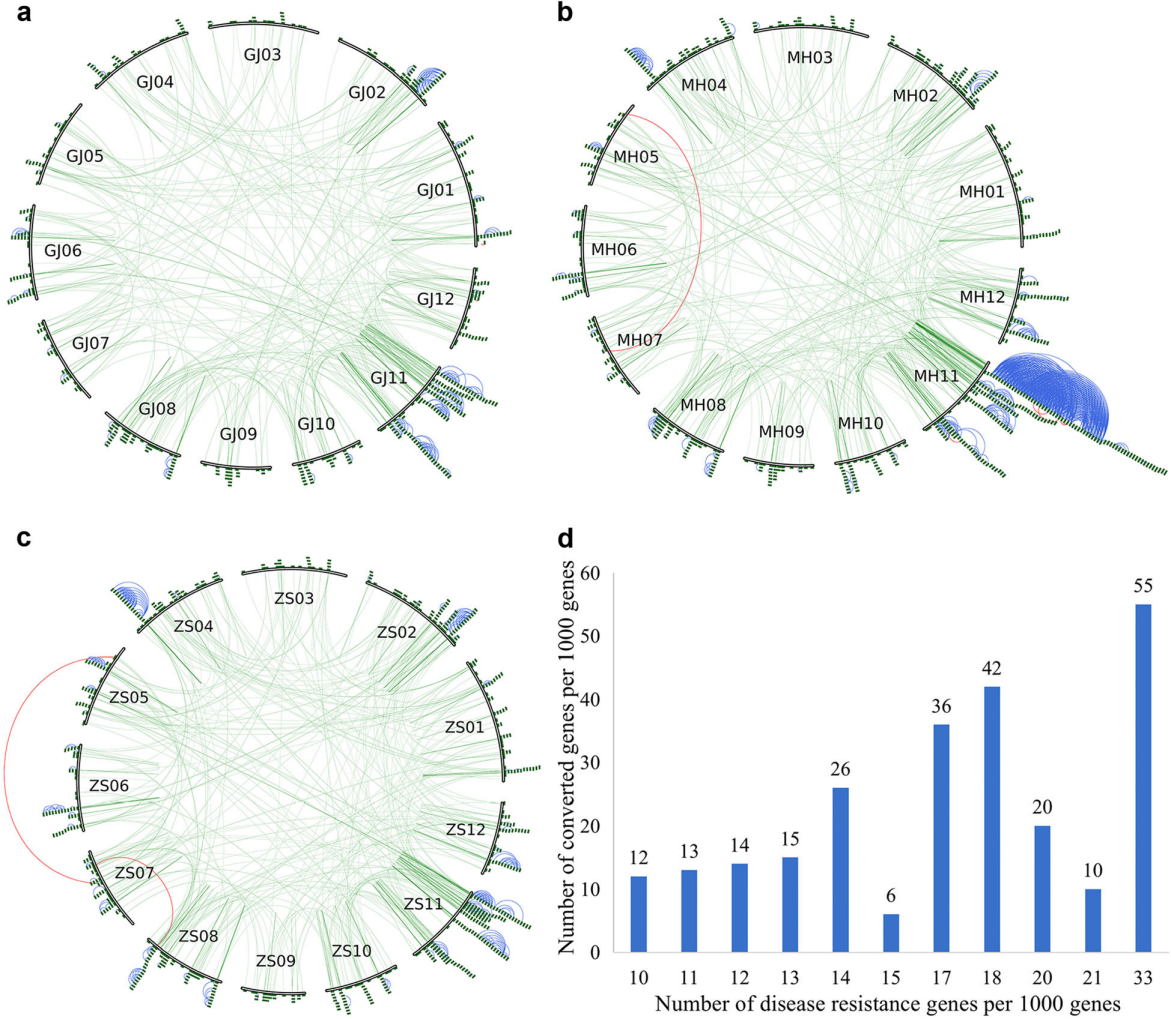
NBS-LRR gene amplification model in three rice subspecies genomes. **a-c** Distribution of NBS-LRR genes on 12 chromosomes in GJ, XI-MH63, and XI-ZS97. Green curved lines within the inner circle connect homologous pairs of NBS-LRR genes on the 12 chromosomes. Green blocks indicate NBS-LRR genes; red lines between NBS-LRR genes indicate *Ks* < 0.1, yellow lines indicate 0.1 < *Ks* < 0.2, and blue lines indicate *Ks* < 1. **d** Relationship between NBS-LRR genes and gene conversion in regions with more than 1% of the NBS-LRR genes in the three genomes

GO analysis of the NBS-LRR genes in the genomes revealed enrichment mainly in terms associated with molecular functions and biological processes (Additional file 16: Fig. S7). In GJ, XI-MH63 and XI-ZS97, 97, 91.1, and 93.1% of genes, respectively, were involved in binding (Additional file 17: Table S10). Therefore, the NBS-LRR genes may be associated with the molecular function of binding and may be biased towards the occurrence of gene conversion. Polyploidization results in expansion of NBS-LRR genes, with ectopic recombination causing the NBS-LRR genes to further undergo a birth-to-death process [37]. Evolutionary analysis of the NBS-LRR genes revealed 25, 67, and 39 younger genes with *Ks* < 0.1 in the three genomes (Fig. 6a-c). Most of these NBS-LRR genes were generated after the divergence of rice subspecies, and clusters of younger NBS-LRR genes were found on chromosomes 2 and 11. These NBS-LRR genes showed a pattern of proximal localization had very recently originated in the three genomes, and were similar to ongoing converted genes distributed at the ends of chromosomes. Furthermore, we found a positive correlation between NBS-LRR genes and converted genes in chromosomal regions with more than 1% of the NBS-LRR genes (Fig. 6d). This suggested that during rice subspecies or genome divergence, the NBS-LRR genes might have contributed to the recombination of homeologous chromosome intervals and indirectly promoted the occurrence of gene conversion.

### The transcriptomic pattern for gene conversion

To analyse the effect of gene conversion on gene expression patterns, we selected transcriptome data from different tissues for three rice genomes and compared the expression differences between converted and nonconverted genes. We found that the number of converted duplicated gene pairs was greater than the number of nonconverted pairs when the difference in expression was greater than twofold (Additional file 18: Table S11). For example, in the panicle of GJ, 60.33% (219/363) of the converted gene pairs had differential expression greater than a twofold change, and he rate of no gene conversion was 62.03% (1493/2407). In the leaves of XI-ZS97, 60.38% (189/313) of converted gene pairs showed differential expression that was more than twofold changed, and the rate of no gene conversion was 70.26% (1304/1856). Furthermore, we found that the expression difference of converted gene pairs was significantly lower than that of nonconverted gene pairs in the three rice genomes (Additional file 19: Table S12; Additional file 20: Table S13). In the panicle of GJ, the average difference in FPKM values of converted gene pairs was 20.76, which was significantly lower than that of nonconverted gene pairs (26.03, *P* = 0.010, t-test). This pattern was also shown in other samples in GJ and in the two XI genomes (Additional file 20: Table S13). From these results, we conclude that the converted genes tend to have more similar expression patterns than the nonconverted duplicates.

## Discussion

### On-going conversion between duplicated genes

Recombination between neohomologous chromosome pairs or homeologous chromosomes resulting from WGD has existed throughout a long evolutionary history and has generated massive chromosomal rearrangements [67–69]. This recombination can persist for a long time, even hundreds of millions of years [70]. Comparisons of rice and sorghum have illustrated that many duplicated genes from WGD events approximately 100 mya are affected by illegitimate recombination and gene conversion events [71, 72]. We compared the GJ, XI-ZS97, and XI-MH63 genomes, revealing extensive whole and partial gene conversion between ~ 100 mya duplicated gene pairs across three rice genomes. The duplicate conversion events deduced here occurred within ~ 0.4 mya after the GJ-XI divergence. As a comparison, we also found that the ~ 100 mya duplicated genes in the genus *Setaria* were affected by recent conversion events (for details, see Additional file 21: Supplemental Text). Therefore, we hypothesize that gene conversion is an ongoing evolutionary event and continues to provide a driving force in genome evolution and genetic innovation.

### Staged recombination inhibition of chromosomes 11 and 12

For some genomic regions, illegitimate recombination and gene conversion persist for millions of years, resulting in homeologous chromosome regions looking similar to very young segmental duplications, especially on chromosomes 11 and 12 of rice [36, 73]. For this reason, it was previously thought that rice chromosomes 11 and 12 share an ~ 3 Mb duplicated DNA segment at the termini of their short arms; this duplication was dated based on *Ks* to ~ 5–7 mya and once suspected to represent a segmental duplication more recent than the WGD common to grasses [20, 74, 75]. The orthologous regions of this recent duplication in *Sorghum bicolor* (SB05 and SB08), *Setaria italica* (SI03, SI07 and SI08), and *Brachypodium distachyon* (BD04) are also likely such an apparently recent duplication in their genomes (Additional file 22: Fig. S8). It would be highly unlikely that each segment duplication occurred independently in such closely corresponding locations in several linkages of reproductive isolation. Previous studies comparing rice with the sorghum genome revealed that rice chromosomes 11 and 12 were most affected by conversion and experienced illegitimate recombination that was temporally restricted in a stepwise manner, producing structural stratification in the chromosomes [28, 29]. Here, we found that chromosomes 11 and 12, especially the duplicated genes located at 2 Mb at the termini of chromosomes, were most affected by recent conversion events in the three *Oryza* genomes. These results further confirm that the illegitimate recombination of rice chromosomes 11 and 12 was staged recombination inhibition. However, this staged recombination inhibition occurred independently in different grass lineages, with the low sequence divergence between paleoduplicated genes preserved in parallel for millions of years since the divergence of these lineages. For example, in *Setaria italica* and *Setaria viridis*, chromosomes 3, 7 and 8 are orthologous to rice chromosomes 11 and 12 (Additional file 22: Fig. S8; Additional file 23: Fig. S9), with duplicated genes also affected by recent conversion. However, the paralogues on chromosomes 3, 7 and 8 of *Setaria italica* and *Setaria viridis* did not show the highest gene conversion rate among the homologous chromosomes (Additional file 24: Table S14). This may be due to specific translocation caused by chromosomal rearrangement in *Setaria* (for details, see Additional file 21: Supplemental Text).

### Gene conversion and physical location

Based on the analysis of the relationship between the gene conversion rate and the distance to telomeres, we found that duplicated genes within the chromosomal termini region are more frequently affected by recent gene conversion in rice or subspecies genomes. Comparing the rice and sorghum genomes, it was shown that more than 50% of wholly converted genes are distributed within the initial 2 Mb regions on their chromosomal termini [28]. As a comparison, we further identified the gene conversion between duplicated genes in *Setaria italica* and *Setaria viridis* and found that the gene conversion rate near the telomere did not appear to be significantly higher than that in other regions of the chromosomes (Additional file 25: Table S15; Additional file 26: Fig. S10). However, based on the division of rice chromosomal regions, we reanalysed the gene conversion rate on the corresponding orthologous regions in the *Setaria italica* and *Setaria viridis* genomes (Additional file 27: Table S16). We also found a similar phenomenon of a higher terminal gene conversion rate. It may be that the genome structure of rice is more conserved, which better maintains the ancestral structure [62]. Therefore, we conclude that duplicated genes are biased towards being affected by conversion near the termini of chromosomes in the rice genome, and in other species with relatively conserved genomic structures, such as sorghum, this phenomenon is also shown [28].

### Gene conversion has contributed to cultivated rice divergence

Gene conversion is the result of recombination. Classical theoretical studies point out that recombination accelerates mutation [72, 76]. Gene conversion may therefore play an important role in species divergence, and we compared the duplicated pairs in *Oryza* genomes to further support this conclusion. We identified that the *Ks* between orthologous genes involved in conversion was significantly smaller than that between orthologous genes not involved in conversion. This suggests that genes affected by gene conversion may have accelerated species evolution, which has been demonstrated by comparison of the duplicated genes in rice and sorghum [33, 77]. In addition, gene conversion can maintain the similarity between paralogues, and gene conversion is based on sequence similarity [78], so the converted paralogues can provide the impetus for further conversion. Our results showed that 46% of ancient gene conversions may have again undergone gene conversion more recently after the divergence of rice subspecies. Therefore, ongoing gene conversion is an accelerating force in the genetic evolution of mutations, and these converted genes may restart the evolutionary process and accelerate the divergence of rice subspecies.

### Gene conversion and chromosome rearrangement

Gene conversion is not necessary for the survival of the species, and most grasses have undergone complex chromosome rearrangements [34, 67]. Our results revealed that chromosome rearrangements are associated with ongoing gene conversion events and may have contributed directly to restricted recombination/duplicated gene conversion between homoeologous regions. Previous studies reported that there was a large inversion in the ancestral chromosome short arm before rice–sorghum divergence, which may suppress gene conversion, resulting in the lowest rate of gene conversion occurring between chromosomes 1 and 5 in rice [28, 44]. However, we did not find the lowest rates of ongoing gene conversion between chromosomes 1 and 5 in the rice subspecies, possibly because chromosome recombination may be a stage-specific evolution event. Here, we found that the two varieties of XI had poor collinearity and had more gene loss than GJ, which may be due to more recent and small-scale genomic rearrangements in their genomes [9]. However, the duplicated genes of XI were more affected by ongoing conversion than those of GJ, implying that DNA damage by recent small-scale chromosome rearrangement could provide conditions for ongoing gene conversion.

### Donor genes are biased towards donating

Gene conversion involves copying one gene sequence from a donor locus to a receptor locus [79]. Analysing the scale of gene conversion helps to illuminate the mechanism of gene conversion [78]. We found that independent conversion events that have survived (thus far) in different lineages have often used the same genes as donors. It seems improbable to attribute this to selection, noting that the donor and acceptor have coexisted in the genome for ~ 100 million years. A more plausible explanation is that one gene copy has some ‘privileged’ nature over the other. This could be genetic or epigenetic. If one gene or its neighbouring region possesses mutations or epigenetic changes, the other gene might be more likely to act as a donor, helping to reinstate intactness. Moreover, some homologous chromosomal segments also seem to be preferential donors rather than acceptors. Mechanisms underlying this bias remain unknown, but an exciting future investigation will be to explore epigenetic phenomena such as patterns that influence gene retention and loss along chromosome segments [80].

### Gene conversion and function

Gene conversion leads to genes similar or even identical in sequence. The analysis above indicates that large gene families may be more susceptible to gene conversion. Duplicated genes may neutralize the presence of putative mutations, providing an opportunity for functional innovation [81]. Rather than being a conservative factor among different genotypes, gene conversion accelerates divergence [29]. Gene conversion has been used to explain the evolution of large gene families, such as NBS-LRR genes and rRNA genes, which typically have dozens of copies on chromosomes [82–84]. Extensive analysis has shown that the evolution of functional genes that are members of large families may often be accompanied by strong purifying selection. Until 1990, most multigene families were thought to have coevolved with related homologous genes through gene conversion [85]. Evolution of the NBS-LRR gene family, rRNA gene family, and some other highly conserved gene families may be consistent with this conclusion. For these families, most genes are usually extremely similar. New genes are created through gene duplication; some of these genes remain in the genome for a long time while others may be lost [86].

### Conversion makes the duplicated gene pairs more similar in expression

Transcriptome sequencing technology has recently been developed to study functional genomics [87]. Increasing transcriptome data provide additional ideas for genome analysis. Differential expression analysis revealed the effect of gene conversion on gene expression patterns. The transcriptome is the link between the genetic information of the genome and the proteome of biological functions, and the expression difference between converted genes and nonconverted genes suggests that gene conversion may have a certain impact on gene function [88]. Here, we detected the differential expression between converted genes and nonconverted genes, implying that the converted genes tend to have more similar expression patterns than nonconverted genes. Gene conversion usually occurs between homologous genes, and the functions of homologous genes are generally similar [36].

## Conclusion

Duplicated genes produced by polyploidization ~ 100 mya were converted in the GJ and XI genomes. By performing comparative genomics and phylogenetic analyses, notably, we showed that ongoing gene conversion has maintained similarity between very ancient duplicates, provided opportunities for further gene conversion, and accelerated rice divergence. Chromosome rearrangements after polyploidization are associated with ongoing gene conversion events, directly restrict recombination and inhibit the duplication of genes between homeologous regions. Our work will contribute to understanding the evolution of duplicated genes affected by recent gene conversion in rice subspecies.

## Supplementary Information

**Additional file 1: Table S1.** Number of homologous genes and blocks within GJ, XI-MH63*,* and XI-ZS97 genomes and between genomes.

**Additional file 2: Table S2.** Identified quartets and gene conversion in GJ*,* XI-MH63, and XI-ZS97.

**Additional file 3: Table S3.** Gene conversion of the three rice subspecies genomes.

**Additional file 4: Fig. S1.** Distribution of amino acid identity between duplicated genes in rice subspecies genomes. **(a)** Amino acid identity distribution of orthologous genes between XI-MH63 and GJ. **(b)** Amino acid identity distribution of orthologous genes between XI-ZS97 and GJ. **(c)** Amino acid identity distribution of orthologous genes between XI-MH63 and XI-ZS97. **(d)** Amino acid identity distribution between paralogous genes in GJ. **(e)** Amino acid identity distribution between paralogous genes in XI-MH63. **(f)** Amino acid identity distribution between paralogous genes in XI-ZS97.

**Additional file 5: Fig. S2.** Distribution of the synonymous nucleotide substitution percentage (Ps) between syntenic paralogues in duplicated blocks of rice subspecies genomes. **(a)** Ps distribution of orthologous genes between XI-MH63 and GJ. **(b)** Ps distribution of orthologous genes between XI-ZS97 and GJ. **(c)** Ps distribution of orthologous genes between XI-MH63 and XI-ZS97. **(d)** Ps distribution between paralogous genes in GJ. **(e)** Ps Distribution between paralogous genes in XI-MH63. **(f)** Ps distribution of between paralogous genes in XI-ZS97.

**Additional file 6: Table S4.** Homology of donor locus and acceptor locus gene conversions.

**Additional file 7: Table S5.** Distribution of converted and nonconverted paralogues in the GJ*,* XI-MH63, and XI-ZS97 genomes.

**Additional file 8: Table S6.** Relationship between the block number and the gene conversion rate in the three rice subspecies genomes.

**Additional file 9: Table S7.** Relationship between the block length and the gene conversion rate in the three rice subspecies genomes.

**Additional file 10: Fig. S3.** Relationship between the length of blocks on each chromosome and the rate of gene conversion. **(a)** The relationship between block length in 12 chromosomes and the gene conversion rate on the corresponding chromosomes of GJ, XI-MH63, and XI-ZS97. **(b)** After removing the four special chromosomes (homologous chromosome pair 1-5 and homologous chromosomes pair 11-12), the relationship between the block length on the 8 chromosomes and the gene conversion rate on the corresponding chromosomes.

**Additional file 11: Fig. S4.** Histogram of Gene Ontology (GO) statistics for converted genes and nonconverted genes in GJ. X-axis shows user selected GO terms; Y-axis shows the percentages of genes (number of a particular gene divided by total gene number).

**Additional file 12: Fig. S5.** Histogram of Gene Ontology (GO) statistics for converted genes and nonconverted genes in *XI-MH63*. X-axis shows user selected GO terms; Y-axis shows the percentages of genes (number of a particular gene divided by total gene number).

**Additional file 13: Fig. S6.** Histogram of Gene Ontology (GO) statistics for converted genes and nonconverted genes in XI-ZS97. X-axis shows user selected GO terms; Y-axis shows the percentages of genes (number of a particular gene divided by total gene number).

**Additional file 14: Table S8.** GO analysis of converted genes and nonconverted genes in GJ*,* XI-MH63, and XI-ZS97.

**Additional file 15: Table S9.** NBS-LRR gene counts by chromosome in GJ*,* XI-MH63, and XI-ZS97.

**Additional file 16: Fig. S7.** Histogram of Gene Ontology (GO) statistics of NBS-LRR genes in GJ, XI-MH63 and XI-ZS97*.* X-axis shows user selected GO terms; Y-axis shows the percentages of genes (number of a particular gene divided by total gene number).

**Additional file 17: Table S10.** GO annotation analysis of NBS-LRR genes in GJ, XI-MH63, and XI-ZS97.

**Additional file 18: Table S11.** Comparison of expression differences between converted and nonconverted gene pairs.

**Additional file 19: Table S12.** Expression level of converted and nonconverted gene pairs in GJ, XI-MH63, and XI-ZS97.

**Additional file 20: Table S13.** Comparison of the mean FPKM difference between converted and nonconverted gene pairs.

**Additional file 21: Supplemental Text.** Gene conversion and occurrence patterns.

**Additional file 22: Fig. S8.** Homologous regions between chromosomes 11 and 12 of GJ with *Sorghum bicolor*, *Setaria italica*, and *Brachypodium distachyon*. The red to blue gradient lines between chromosomes connect paralogous genes, and colors corresponding to *Ks* values. The gray lines connect the orthologous genes.

**Additional file 23: Fig. S9.** Homologous dot plot between GJ and *Setaria italica*. The best, secondary, and other matched homologous gene pairs output by Blast were dotploted by red, blue, and gray colors in this figure.

**Additional file 24: Table S14.** Distribution of paralogues and converted genes pairs in *Setaria italica* and *Setaria viridis*.

**Additional file 25: Table S15.** Relationship between gene physical location and gene conversion in *Setaria italica* and *Setaria viridis*.

**Additional file 26: Fig. S10.** Conversion patterns in *Setaria italica* and *Setaria viridis*. **(a)** Gene conversion in *Setaria italica.* **(b)** Gene conversion in *Setaria viridis.* The lines in the circle represent the gene conversion.

**Additional file 27: Table S16.** Relationship between gene physical location and gene conversion of *Setaria italica* and *Setaria viridis* in rice orthologous regions.

## Data Availability

The datasets supporting the conclusions of this article are included within the article and its additional files.
