# Supplementary material for: Conversion between 100-million-year-old duplicated genes contributes to rice subspecies divergence
Source: BMC Genomics. 2021 Jun 19;22:460. doi: 10.1186/s12864-021-07776-y (PMC8214281; doi:10.1186/s12864-021-07776-y)
Supplement: Supplementary file 1 — Additional file 1: Table S1. Number of homologous genes and blocks within GJ, XI-MH63, and XI-ZS97 genomes and between genomes. [file 12864_2021_7776_MOESM1_ESM.docx]

**Table S1** Number of homologous genes and blocks within GJ, XI-MH63*,* and XI-ZS97 genomes and between genomes.

| **Intra/intergenomic** | **Block_lens >4** | **Block_lens >10** | **Block_lens >20** | **Block_lens >50** | **ACGP** | **LDB** | **LDB on chromosomes** |
| --- | --- | --- | --- | --- | --- | --- | --- |
| GJ | 292/3314^a^ | 46/1996 | 18/1628 | 10/1373 | 11.35, 43.39, 90.44, 137.3 | 263 | Oj02-Oj04 |
|  | 5251^b^ | 3807 | 3253 | 2746 |  |  |  |
| XI-MH63 | 260/2629 | 35/1406 | 19/1187 | 10/921 | 10.11, 40.17, 62.47, 92.1 | 156 | Mh11-Mh12 |
|  | 4228 | 2761 | 2373 | 1842 |  |  |  |
| XI-ZS97 | 284/2889 | 43/1601 | 17/1225 | 9/947 | 10.17, 37.23, 72.06, 105.22 | 187 | Zs11-Zs12 |
|  | 4572 | 3052 | 2436 | 1894 |  |  |  |
| XI-MH63 *vs*. GJ | 861/26559 | 144/22725 | 79/21902 | 59/21251 | 30.85, 157.81, 277.24, 360.19 | 2305 | Mh02-Oj02 |
|  | 19471 *vs.* 19821 | 19045 *vs*. 19319 | 18948 *vs*. 19178 | 18705 *vs*. 18909 |  |  |  |
| XI-ZS97 *vs*. GJ | 872/25990 | 156/22171 | 84/21232 | 60/20473 | 29.81, 142.12, 252.76, 341.22 | 1633 | Zs01-Oj01 |
|  | 18823 *vs.* 19311 | 18405 *vs.* 18688 | 18280 *vs.* 18508 | 18050 *vs.* 18261 |  |  |  |
| XI-ZS97 *vs*. XI-MH63 | 957/32663 | 160/28473 | 90/27546 | 66/26741 | 34.13, 177.96, 306.07, 405.17 | 2267 | Zs02-Mh02 |
|  | 25675 *vs.* 25784 | 25151 *vs.* 25158 | 25027 *vs.* 25038 | 24813 *vs.* 24828 |  |  |  |

Note: Block_lens: duplicated gene pairs involved in blocks; ACGP: average collinear gene pairs respectively per block; LDB: number of collinear gene pairs reside in the longest duplicated block; ^a^The slash is preceded by the number of blocks, followed by the number of collinear gene pairs; ^b^Duplicated genes involved in blocks.
