# Supplementary material for: Conversion between 100-million-year-old duplicated genes contributes to rice subspecies divergence
Source: BMC Genomics. 2021 Jun 19;22:460. doi: 10.1186/s12864-021-07776-y (PMC8214281; doi:10.1186/s12864-021-07776-y)
Supplement: Supplementary file 2 — Additional file 2: Table S2. Identified quartets and gene conversion in GJ, XI-MH63, and XI-ZS97. [file 12864_2021_7776_MOESM2_ESM.docx]

**Table S2** Identified quartets and gene conversion in GJ*,* XI-MH63, and XI-ZS97*.*

| **Chromosomes**  of paralogues | **Quartets** | Converted paralogues in A | | | | Converted paralogs in B | | | |
| --- | --- | --- | --- | --- | --- | --- | --- | --- | --- |
|  |  | **WCV-I^1^** | **WCV-II^2^** | **PCV^3^** | **Total** | **WCV-I^1^** | **WCV-II^2^** | **PCV^3^** | **Total** |
| **XI-ZS97 *vs*. GJ.** | | | | | | | | | |
| chr01-chr05 | 791 | 2 (0.25%） | 40 (5.06%) | 74 (9.36%) | 116 (14.66%) | 2 (0.25%) | 31 (3.92%) | 54 (6.83%) | 87 (11.00%) |
| chr02-chr06 | 431 | 0 | 13 (3.02%) | 38 (8.82%) | 51 (11.83%) | 0 | 10 (2.32%) | 10 (2.32%) | 20 (4.64%) |
| chr02-chr04 | 379 | 0 | 26 (6.86%) | 32 (8.44%) | 58 (15.30%) | 0 | 18 (4.75%) | 23 (6.07%) | 41 (10.82%) |
| chr03-chr10 | 242 | 0 | 15 (6.20%) | 22 (9.09%) | 37 (15.29%) | 0 | 11 (4.55%) | 18 (7.44%) | 29 (11.98%) |
| chr03-chr07 | 332 | 0 | 14 (4.22%) | 31 (9.34%) | 45 (13.55%) | 0 | 10 (3.01%) | 28 (8.43%) | 38 (11.45%) |
| chr03-chr12 | 83 | 0 | 5 (6.02%) | 7 (8.43%) | 12 (14.46%) | 0 | 3 (3.61%) | 4 (4.82%) | 7 (8.43%) |
| chr04-chr08 | 51 | 0 | 4 (7.84%) | 6 (11.76%) | 10 (19.61%) | 0 | 1 (1.96%) | 1 (1.96%) | 2 (3.92%) |
| chr08-chr09 | 281 | 1 (0.36%) | 12 (4.27%) | 27 (9.61%) | 40 (14.23%) | 2 (0.71%) | 10 (3.56%) | 16 (5.69%) | 28 (9.96%) |
| chr11-chr12 | 289 | 4 (1.73%) | 46 (15.92%) | 10 (3.46%) | 61 (21.11%) | 8 (3.11%) | 37 (12.80%) | 21 (7.27%) | 67 (23.18%) |
| summary | 2879 | 7 | 175 | 247 | 429 | 12 | 131 | 175 | 318 |
| **XI-MH63 *vs*. GJ** | | | | | | | | | |
| chr01-chr05 | 675 | 1 (0.15%) | 33 (4.89%) | 63 (9.33%) | 97 (14.37%) | 1 (0.44%) | 30 (4.44%) | 37 (5.48%) | 70 (10.37%) |
| chr02-chr06 | 418 | 0 | 17 (4.07%) | 49 (11.72%) | 66 (15.79%) | 0 | 11 (2.63%) | 24 (5.74%) | 35 (8.37%) |
| chr02-chr04 | 380 | 0 | 25 (6.58%) | 35 (9.21%) | 60 (15.79%) | 0 | 17 (4.47%) | 18 (4.74%) | 35 (9.21%) |
| chr03-chr10 | 227 | 0 | 13 (5.73%) | 21 (9.25%) | 34 (14.98%) | 0 | 11 (4.85%) | 15 (6.61%) | 26 (11.45%) |
| chr03-chr07 | 391 | 0 | 16 (4.09%) | 37 (9.46%) | 53 (13.55%) | 0 | 10 (2.56%) | 28 (7.16%) | 38 (9.72%) |
| chr03-chr12 | 103 | 0 | 4 (3.88%) | 10 (9.71%) | 14 (13.59%) | 0 | 3 (2.91%) | 7 (6.80%) | 10 (9.71%) |
| chr04-chr08 | 48 | 0 | 4 (8.33%) | 2 (4.17%) | 6 (12.50%) | 0 | 2 (4.17%) | 2 (4.17%) | 4 (8.33%) |
| chr08-chr09 | 249 | 0 | 11 (4.42%) | 25 (10.04%) | 36 (14.46%) | 0 | 10 (4.02%) | 24 (9.64%) | 34 (13.65%) |
| chr11-chr12 | 297 | 6 (2.69%) | 50 (16.84%) | 12 (4.04%) | 70 (23.57%) | 5 (2.02%) | 36 (12.12%) | 13 (4.38%) | 55 (18.52%) |
| summary | 2788 | 7 | 173 | 254 | 434 | 6 | 130 | 168 | 304 |
| **XI-ZS97 *vs*. XI-MH63** | | | | | | | | | |
| chr01-chr05 | 700 | 0 | 0 | 26 (3.71%) | 26 (3.71%) | 0 | 0 | 17 (2.43%) | 17 (2.43%) |
| chr02-chr06 | 427 | 0 | 1 (0.23%) | 11 (2.58%) | 12 (2.81%) | 0 | 0 | 12 (2.81%) | 12 (2.81%) |
| chr02-chr04 | 357 | 0 | 0 | 6 (1.68%) | 6 (1.68%) | 0 | 0 | 8 (2.24%) | 8 (2.24%) |
| chr03-chr10 | 240 | 0 | 0 | 8 (3.33%) | 8 (3.33%) | 0 | 0 | 9 (3.75%) | 9 (3.75%) |
| chr03-chr07 | 268 | 0 | 0 | 13 (4.85%) | 13 (4.85%) | 0 | 0 | 12 (4.48%) | 12 (4.48%) |
| chr03-chr12 | 21 | 0 | 0 | 0 | 0 | 0 | 0 | 0 | 0 |
| chr04-chr08 | 0 | 0 | 0 | 0 | 0 | 0 | 0 | 0 | 0 |
| chr08-chr09 | 282 | 0 | 0 | 6 (2.13%) | 6 (2.13%) | 0 | 0 | 3 (1.06%) | 3 (1.06%) |
| chr11-chr12 | 271 | 1 (0.37%) | 1 (0.37%) | 7 (2.58%) | 9 (3.32%) | 1 (0.37%) | 1 (0.37%) | 16 (5.90%) | 18 (6.64%) |
| Summary | 2566 | 1 | 2 | 77 | 80 | 1 | 1 | 77 | 79 |

Note**: ^1^**Wholly converted paralogues inferred by Ks value as a measurement.

^2^Wholly converted paralogues inferred by counted the identical sites.

^3^Partially converted paralogues
