# Supplementary figures and images for: Conversion between 100-million-year-old duplicated genes contributes to rice subspecies divergence
Source: BMC Genomics. 2021 Jun 19;22:460. doi: 10.1186/s12864-021-07776-y (PMC8214281; doi:10.1186/s12864-021-07776-y)

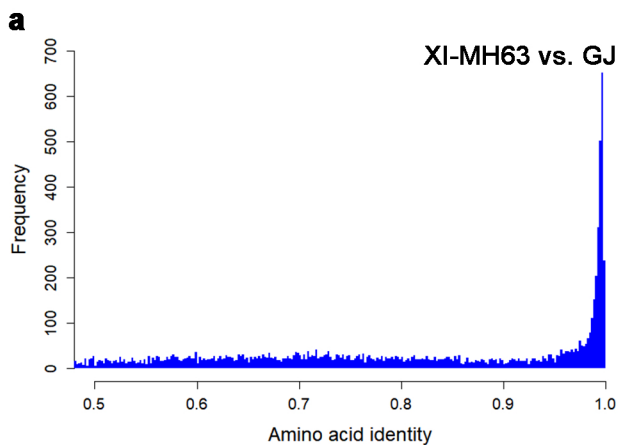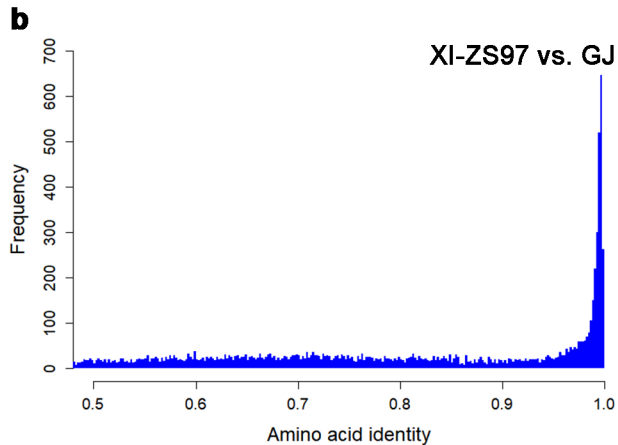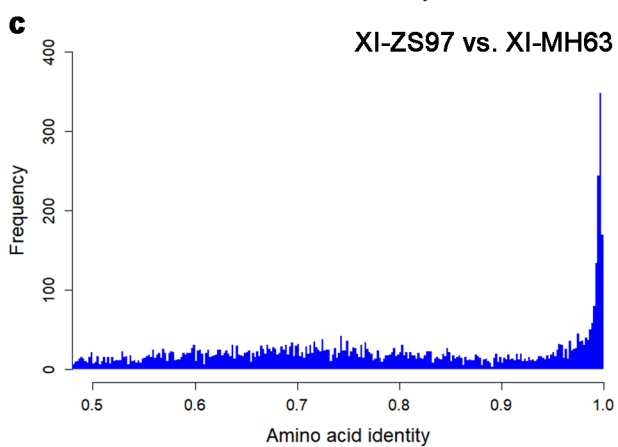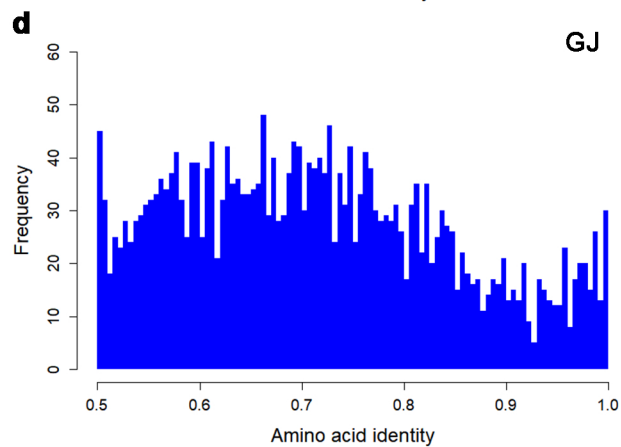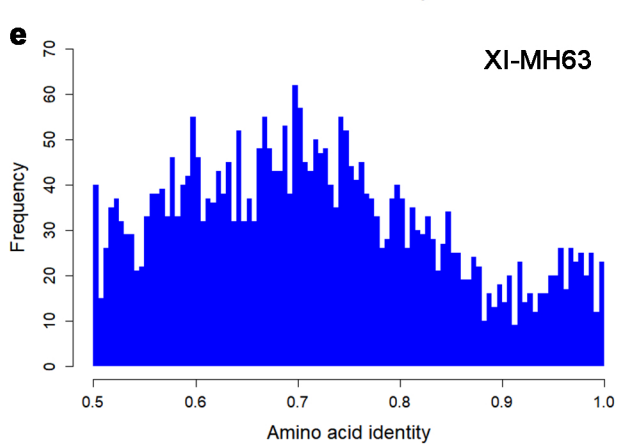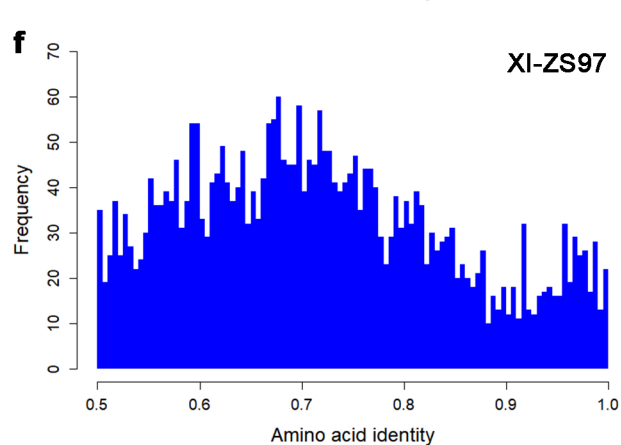

Supplement: Supplementary file 4 — Additional file 4: Fig. S1. Distribution of amino acid identity between duplicated genes in rice subspecies genomes. (a) Amino acid identity distribution of orthologous genes between XI-MH63 and GJ. (b) Amino acid identity distribution of orthologous genes between XI-ZS97 and GJ. (c) Amino acid identity distribution of orthologous genes between XI-MH63 and XI-ZS97. (d) Amino acid identity distribution between paralogous genes in GJ. (e) Amino acid identity distribution between paralogous genes in XI-MH63. (f) Amino acid identity distribution between paralogous genes in XI-ZS97. [file 12864_2021_7776_MOESM4_ESM.pdf]

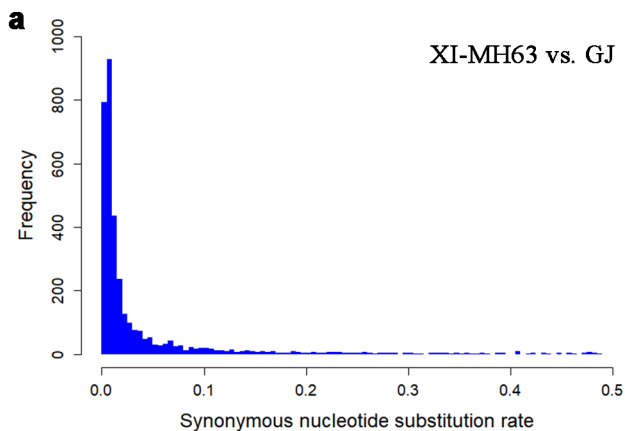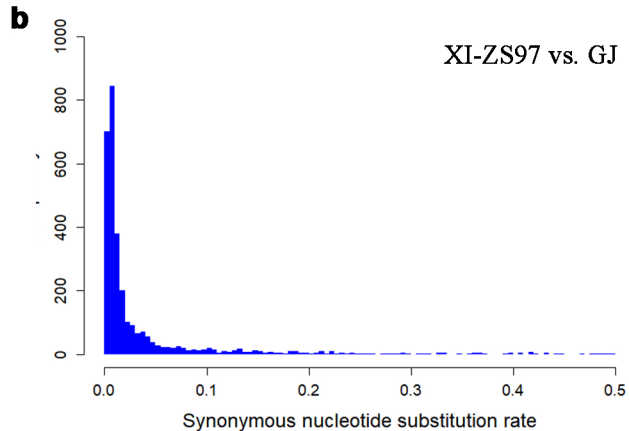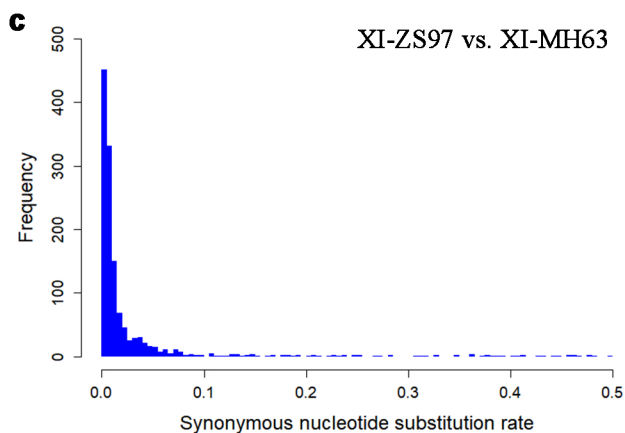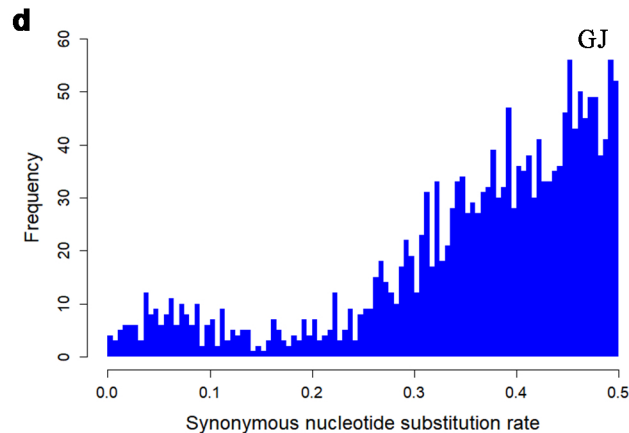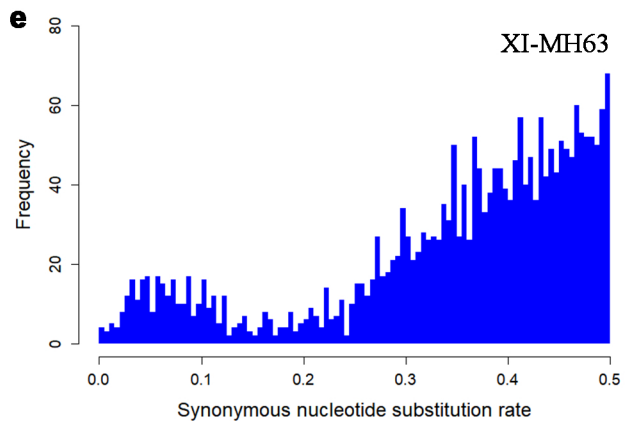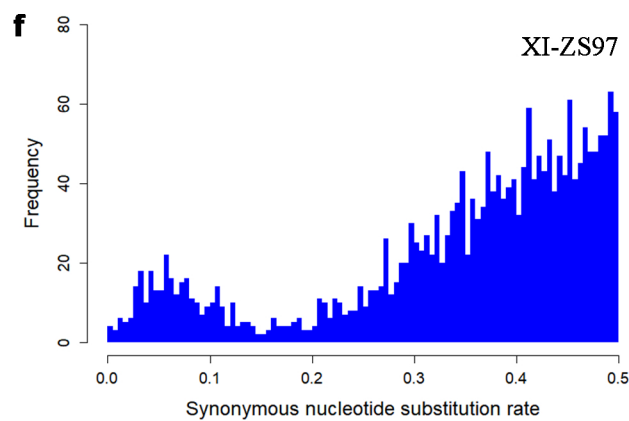

Supplement: Supplementary file 5 — Additional file 5: Fig. S2. Distribution of the synonymous nucleotide substitution percentage (Ps) between syntenic paralogues in duplicated blocks of rice subspecies genomes. (a) Ps distribution of orthologous genes between XI-MH63 and GJ. (b) Ps distribution of orthologous genes between XI-ZS97 and GJ. (c) Ps distribution of orthologous genes between XI-MH63 and XI-ZS97. (d) Ps distribution between paralogous genes in GJ. (e) Ps Distribution between paralogous genes in XI-MH63. (f) Ps distribution of between paralogous genes in XI-ZS97. [file 12864_2021_7776_MOESM5_ESM.pdf]

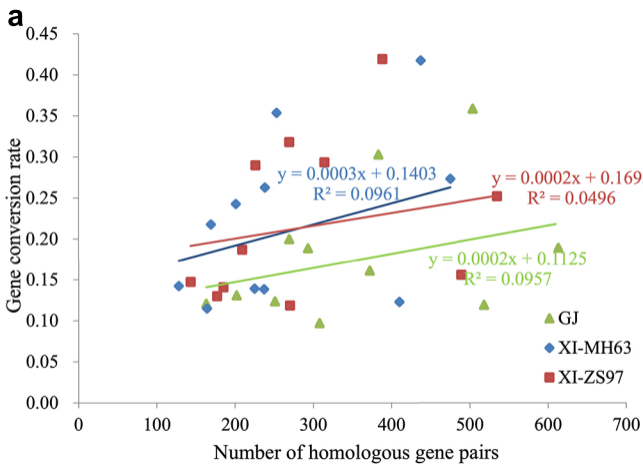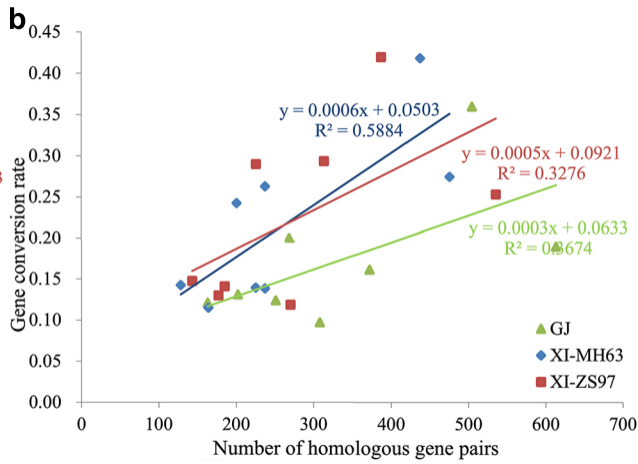

Supplement: Supplementary file 10 — Additional file 10: Fig. S3. Relationship between the length of blocks on each chromosome and the rate of gene conversion. (a) The relationship between block length in 12 chromosomes and the gene conversion rate on the corresponding chromosomes of GJ, XI-MH63, and XI-ZS97. (b) After removing the four special chromosomes (homologous chromosome pair 1-5 and homologous chromosomes pair 11-12), the relationship between the block length on the 8 chromosomes and the gene conversion rate on the corresponding chromosomes. [file 12864_2021_7776_MOESM10_ESM.pdf]

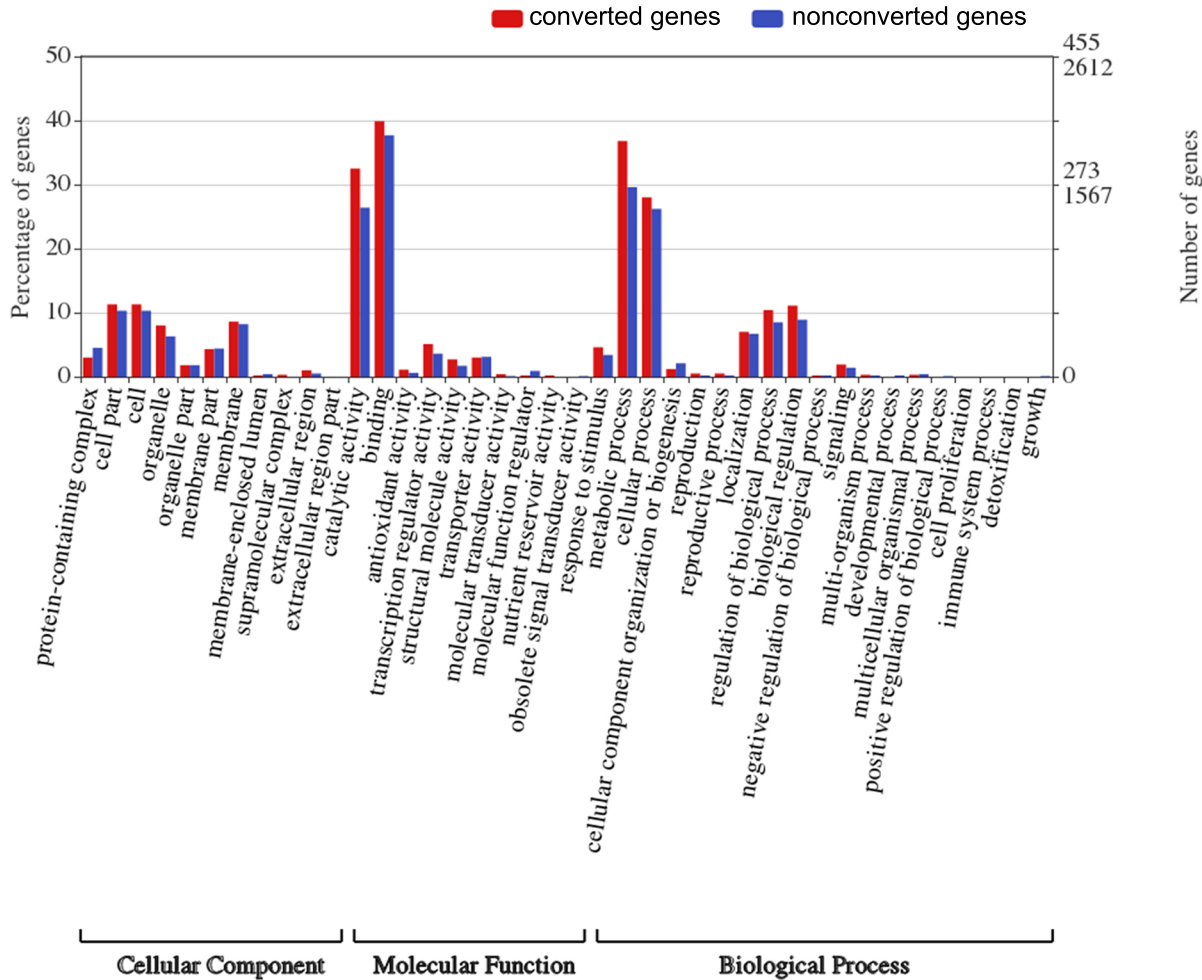

Supplement: Supplementary file 11 — Additional file 11: Fig. S4. Histogram of Gene Ontology (GO) statistics for converted genes and nonconverted genes in GJ. X-axis shows user selected GO terms; Y-axis shows the percentages of genes (number of a particular gene divided by total gene number). [file 12864_2021_7776_MOESM11_ESM.pdf]

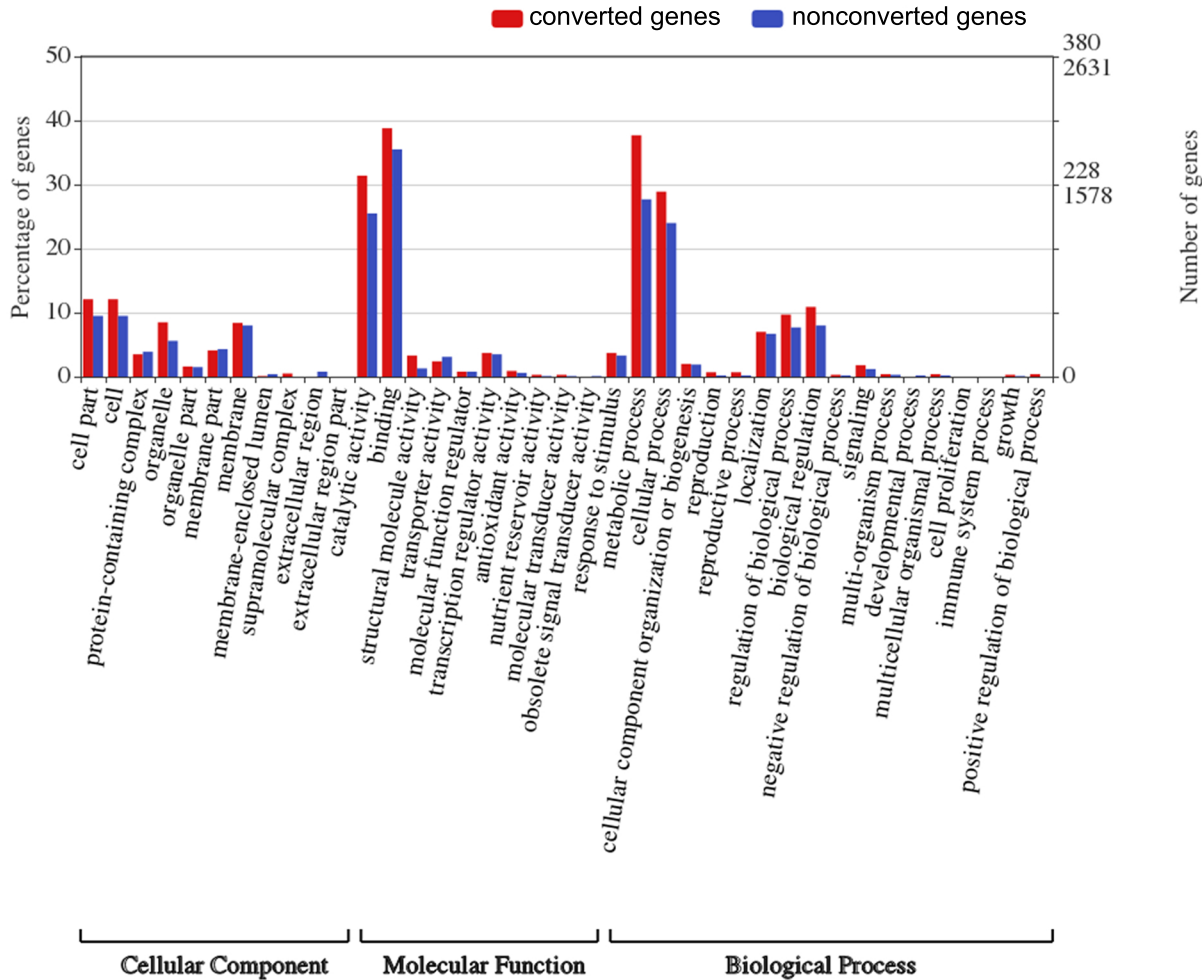

Supplement: Supplementary file 12 — Additional file 12: Fig. S5. Histogram of Gene Ontology (GO) statistics for converted genes and nonconverted genes in XI-MH63. X-axis shows user selected GO terms; Y-axis shows the percentages of genes (number of a particular gene divided by total gene number). [file 12864_2021_7776_MOESM12_ESM.pdf]

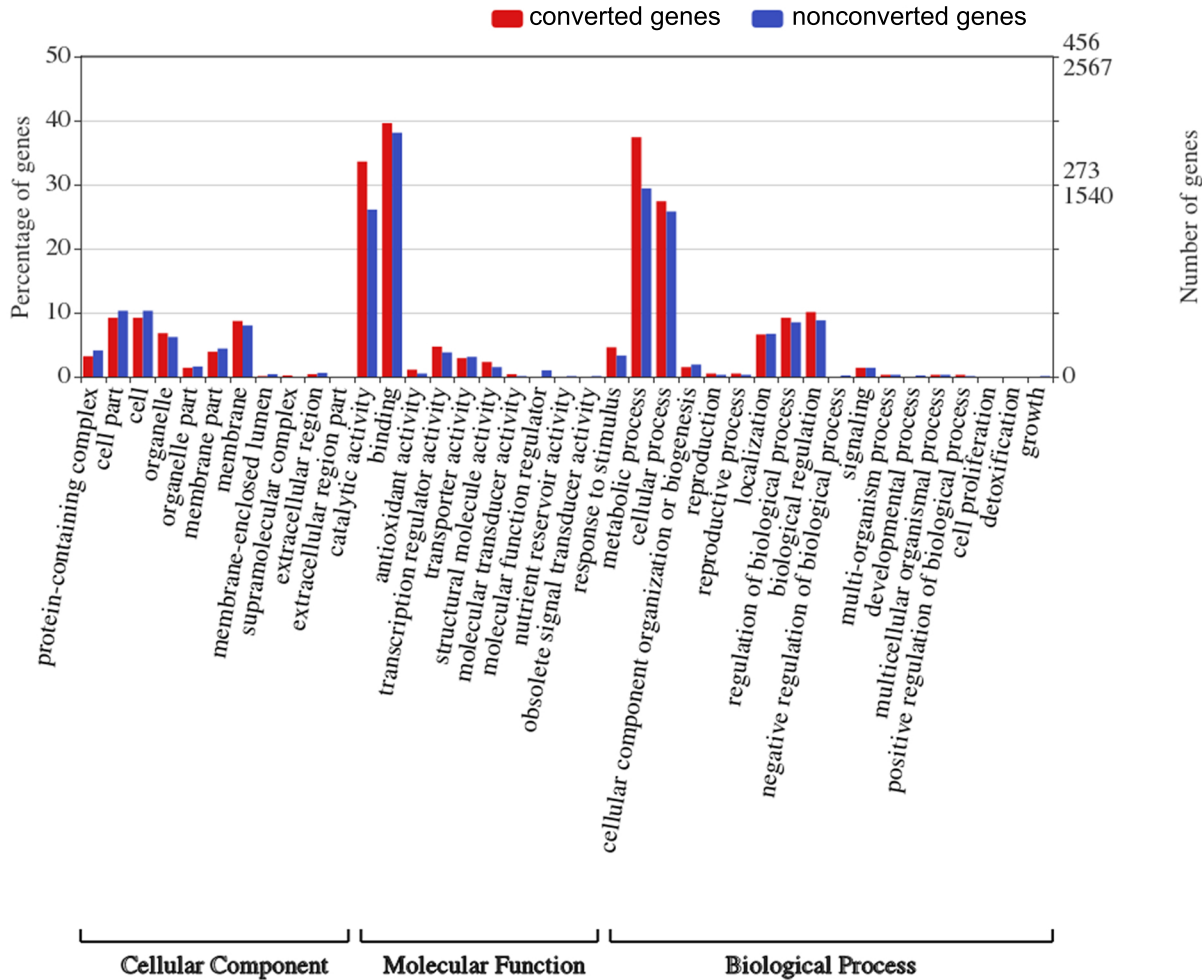

Supplement: Supplementary file 13 — Additional file 13: Fig. S6. Histogram of Gene Ontology (GO) statistics for converted genes and nonconverted genes in XI-ZS97. X-axis shows user selected GO terms; Y-axis shows the percentages of genes (number of a particular gene divided by total gene number). [file 12864_2021_7776_MOESM13_ESM.pdf]

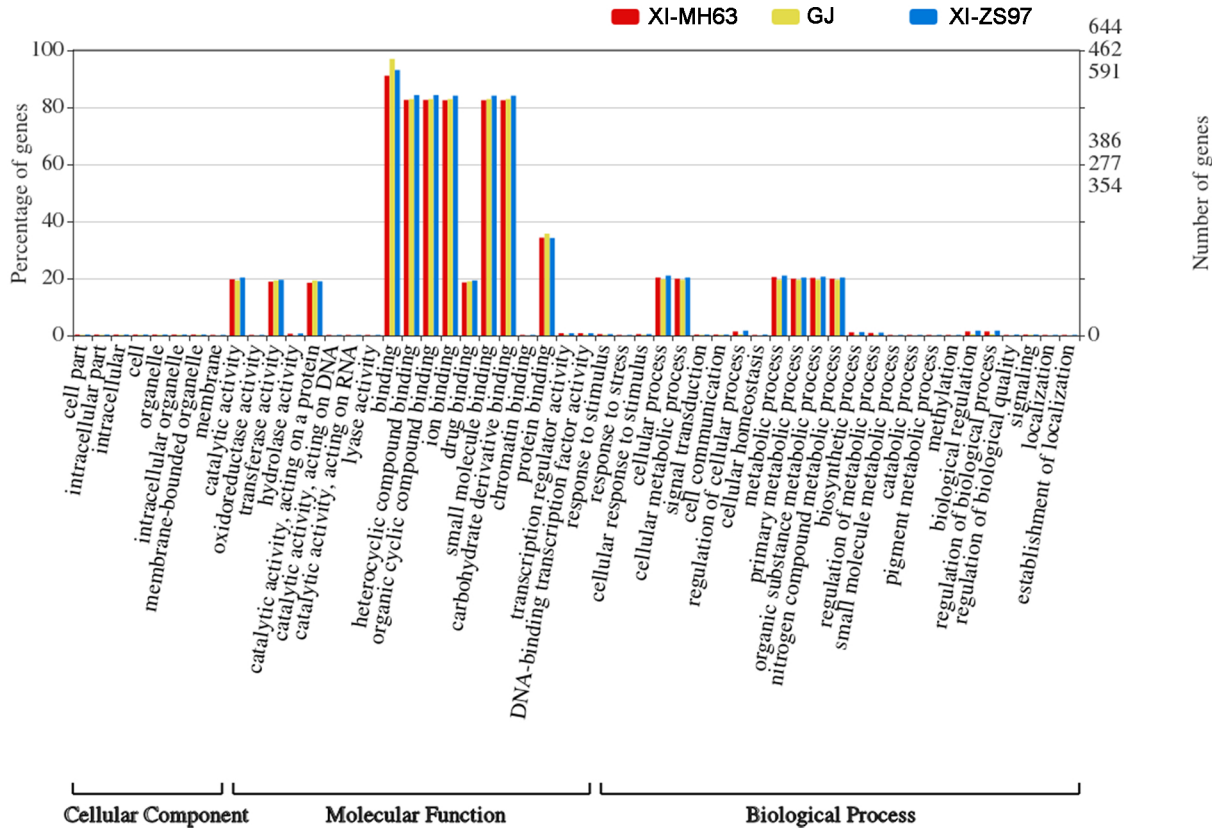

Supplement: Supplementary file 16 — Additional file 16: Fig. S7. Histogram of Gene Ontology (GO) statistics of NBS-LRR genes in GJ, XI-MH63 and XI-ZS97. X-axis shows user selected GO terms; Y-axis shows the percentages of genes (number of a particular gene divided by total gene number). [file 12864_2021_7776_MOESM16_ESM.pdf]

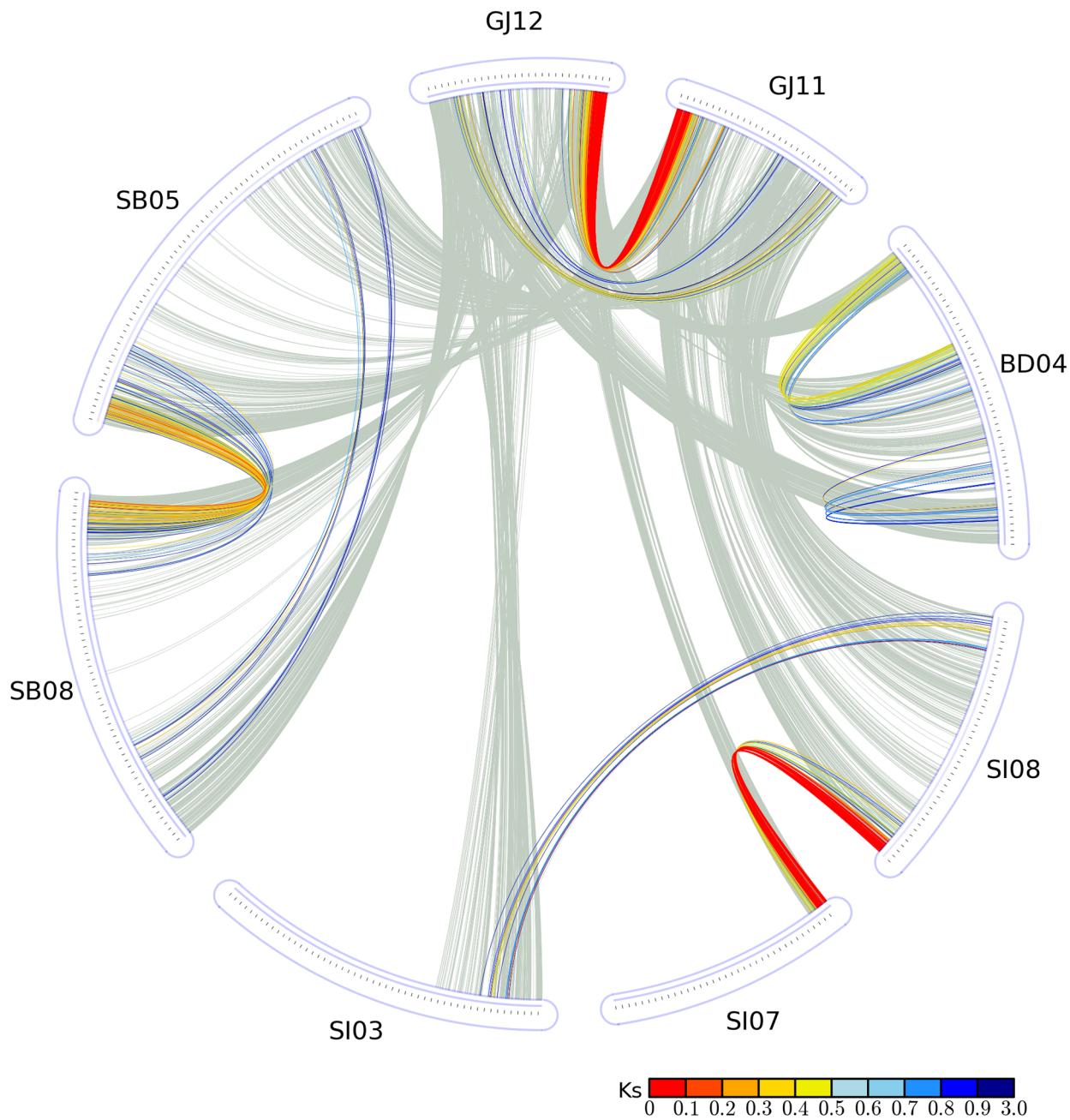

Supplement: Supplementary file 22 — Additional file 22: Fig. S8. Homologous regions between chromosomes 11 and 12 of GJ with Sorghum bicolor, Setaria italica, and Brachypodium distachyon. The red to blue gradient lines between chromosomes connect paralogous genes, and colors corresponding to Ks values. The gray lines connect the orthologous genes. [file 12864_2021_7776_MOESM22_ESM.pdf]

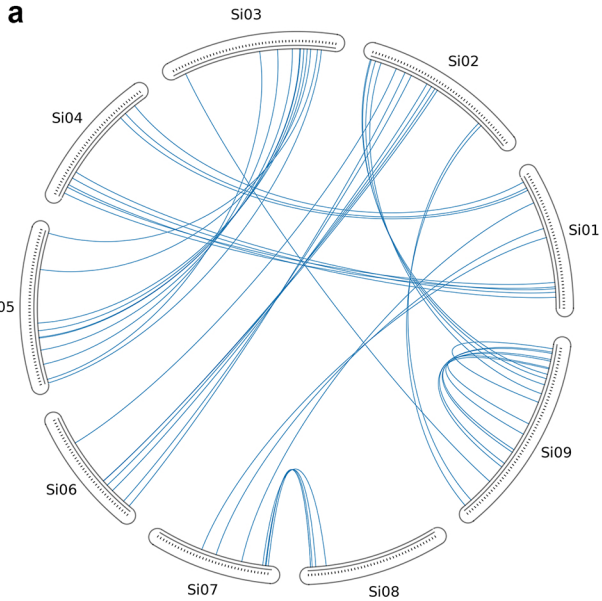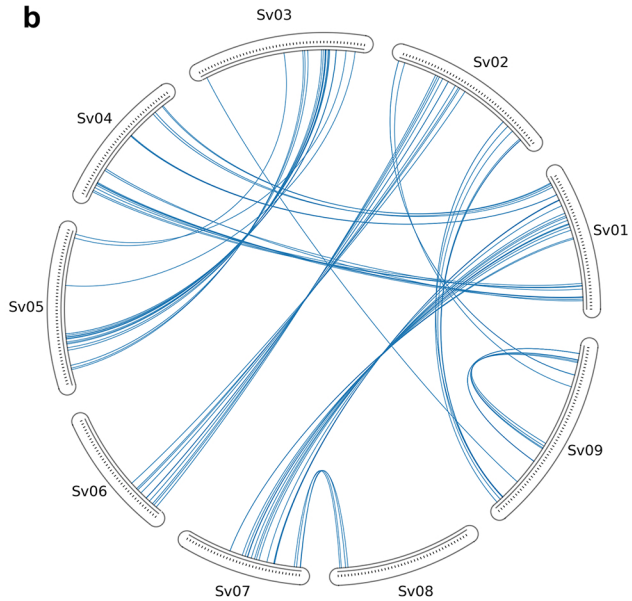

Supplement: Supplementary file 26 — Additional file 26: Fig. S10. Conversion patterns in Setaria italica and Setaria viridis. (a) Gene conversion in Setaria italica. (b) Gene conversion in Setaria viridis. The lines in the circle represent the gene conversion. [file 12864_2021_7776_MOESM26_ESM.pdf]
