# Supplementary material for: Conversion between 100-million-year-old duplicated genes contributes to rice subspecies divergence
Source: BMC Genomics. 2021 Jun 19;22:460. doi: 10.1186/s12864-021-07776-y (PMC8214281; doi:10.1186/s12864-021-07776-y)
Supplement: Supplementary file 7 — Additional file 7: Table S5. Distribution of converted and nonconverted paralogues in the GJ, XI-MH63, and XI-ZS97 genomes. [file 12864_2021_7776_MOESM7_ESM.docx]

**Table S5.** Distribution of converted and nonconverted paralogues in the GJ*,* XI-MH63, and XI-ZS97 genomes*.*

| **Paralogous chromosomes** | **GJ** | | **XI-MH63** | | **XI-ZS97** | |
| --- | --- | --- | --- | --- | --- | --- |
|  | **Paralogues** | **Converted pairs** | **Paralogues** | **Converted pairs** | **Paralogues** | **Converted pairs** |
| chr01-chr05 | 854 | 102(11.94%) | 822 | 101(12.29%) | 845 | 132(15.62%) |
| chr02-chr06 | 505 | 49(9.70%) | 513 | 71(13.84%) | 506 | 60(11.86%) |
| chr02-chr04 | 456 | 42(9.21%) | 444 | 60(13.51%) | 442 | 59(13.35%) |
| chr03-chr10 | 273 | 33(12.09%) | 281 | 40(14.23%) | 285 | 42(14.74%) |
| chr03-chr07 | 420 | 52(12.38%) | 438 | 61(13.93%) | 369 | 52(14.09%) |
| chr03-chr12 | 114 | 13(11.40%) | 103 | 14(13.59%) | 84 | 11(13.10%) |
| chr04-chr08 | 58 | 4(6.90%) | 47 | 6(12.77%) | 50 | 8(16.00%) |
| chr08-chr09 | 313 | 41(13.10%) | 322 | 37(11.49%) | 331 | 43(12.99%) |
| chr11-chr12 | 339 | 64(18.88%) | 349 | 76(21.78%) | 342 | 64(18.71%) |
| Toal | 3332 | 398(12.00%) | 3319 | 466(14.04%) | 3254 | 468(14.47%) |
