# Supplementary material for: Conversion between 100-million-year-old duplicated genes contributes to rice subspecies divergence
Source: BMC Genomics. 2021 Jun 19;22:460. doi: 10.1186/s12864-021-07776-y (PMC8214281; doi:10.1186/s12864-021-07776-y)
Supplement: Supplementary file 8 — Additional file 8: Table S6. Relationship between the block number and the gene conversion rate in the three rice subspecies genomes. [file 12864_2021_7776_MOESM8_ESM.docx]

**Table S6.** Relationship between the block number and the gene conversion rate in the three rice subspecies genomes.

| **Chromosome** | **GJ** | | **XI-MH63** | | **XI-ZS97** | |
| --- | --- | --- | --- | --- | --- | --- |
|  | **Block num** | **CV rate** | **Block num** | **CV rate** | **Block num** | **CV rate** |
| 1 | 19 | 0.12 | 22 | 0.12 | 26 | 0.16 |
| 2 | 18 | 0.19 | 19 | 0.27 | 24 | 0.25 |
| 3 | 28 | 0.36 | 34 | 0.42 | 31 | 0.42 |
| 4 | 16 | 0.16 | 15 | 0.26 | 19 | 0.29 |
| 5 | 19 | 0.12 | 22 | 0.12 | 26 | 0.16 |
| 6 | 10 | 0.10 | 10 | 0.14 | 13 | 0.12 |
| 7 | 8 | 0.12 | 10 | 0.14 | 10 | 0.14 |
| 8 | 11 | 0.20 | 11 | 0.24 | 14 | 0.29 |
| 9 | 3 | 0.13 | 5 | 0.11 | 6 | 0.13 |
| 10 | 11 | 0.12 | 13 | 0.14 | 12 | 0.15 |
| 11 | 4 | 0.19 | 3 | 0.22 | 5 | 0.19 |
| 12 | 13 | 0.30 | 14 | 0.35 | 14 | 0.32 |
