# Supplementary material for: Conversion between 100-million-year-old duplicated genes contributes to rice subspecies divergence
Source: BMC Genomics. 2021 Jun 19;22:460. doi: 10.1186/s12864-021-07776-y (PMC8214281; doi:10.1186/s12864-021-07776-y)
Supplement: Supplementary file 9 — Additional file 9: Table S7. Relationship between the block length and the gene conversion rate in the three rice subspecies genomes. [file 12864_2021_7776_MOESM9_ESM.docx]

**Table S7.** Relationship between the block length and the gene conversion rate in the three rice subspecies genomes.

| **Chromosome** | **GJ** | | **XI-MH63** | | **XI-ZS97** | |
| --- | --- | --- | --- | --- | --- | --- |
|  | **Block len** | **CV rate** | **Block len** | **CV rate** | **Block len** | **CV rate** |
| 1 | 518 | 0.12 | 410 | 0.12 | 489 | 0.16 |
| 2 | 613 | 0.19 | 475 | 0.27 | 535 | 0.25 |
| 3 | 504 | 0.36 | 437 | 0.42 | 388 | 0.42 |
| 4 | 372 | 0.16 | 238 | 0.26 | 314 | 0.29 |
| 5 | 518 | 0.12 | 410 | 0.12 | 489 | 0.16 |
| 6 | 308 | 0.10 | 237 | 0.14 | 270 | 0.12 |
| 7 | 251 | 0.12 | 225 | 0.14 | 185 | 0.14 |
| 8 | 269 | 0.20 | 201 | 0.24 | 226 | 0.29 |
| 9 | 202 | 0.13 | 164 | 0.11 | 177 | 0.13 |
| 10 | 163 | 0.12 | 128 | 0.14 | 143 | 0.15 |
| 11 | 293 | 0.19 | 169 | 0.22 | 209 | 0.19 |
| 12 | 383 | 0.30 | 253 | 0.35 | 269 | 0.32 |
