# Supplementary material for: Conversion between 100-million-year-old duplicated genes contributes to rice subspecies divergence
Source: BMC Genomics. 2021 Jun 19;22:460. doi: 10.1186/s12864-021-07776-y (PMC8214281; doi:10.1186/s12864-021-07776-y)
Supplement: Supplementary file 15 — Additional file 15: Table S9. NBS-LRR gene counts by chromosome in GJ, XI-MH63, and XI-ZS97. [file 12864_2021_7776_MOESM15_ESM.docx]

**Table S9.** NBS-LRR gene counts by chromosome in GJ*,* XI-MH63, and XI-ZS97.

| **Chromosome** | **GJ** | **XI-MH63** | **XI-ZS97** |
| --- | --- | --- | --- |
| 1 | 46(9.96%) | 47(7.30%) | 54(9.14%) |
| 2 | 41(8.87%) | 60(9.32%) | 63(10.66%) |
| 3 | 17(3.68%) | 26(4.04%) | 22(3.72%) |
| 4 | 28(6.06%) | 50(7.76%) | 50(8.46%) |
| 5 | 21(4.55%) | 28(4.35%) | 33(5.58%) |
| 6 | 39(8.44%) | 43(6.68%) | 43(7.28%) |
| 7 | 24(5.19%) | 31(4.81%) | 38(6.43%) |
| 8 | 44(9.52%) | 56(8.70%) | 58(9.81%) |
| 9 | 21(4.55%) | 28(4.35%) | 23(3.89%) |
| 10 | 32(6.93%) | 43(6.68%) | 40(6.77%) |
| 11 | 113(24.46%) | 181(28.11%) | 126(21.32%) |
| 12 | 36(7.79%) | 51(7.92%) | 41(6.94%) |
| Sum | 462 | 644 | 591 |
