# Supplementary material for: Conversion between 100-million-year-old duplicated genes contributes to rice subspecies divergence
Source: BMC Genomics. 2021 Jun 19;22:460. doi: 10.1186/s12864-021-07776-y (PMC8214281; doi:10.1186/s12864-021-07776-y)
Supplement: Supplementary file 17 — Additional file 17: Table S10. GO annotation analysis of NBS-LRR genes in GJ, XI-MH63, and XI-ZS97. [file 12864_2021_7776_MOESM17_ESM.docx]

**Table S10.** GO annotation analysis of NBS-LRR genes in GJ, XI-MH63, and XI-ZS97*.*

| **GO level1** | **GO level2** | **All genes of three genomes which have GO terms^1^** | **Percentage of three genomes which have GO terms^1^** | **P-value** |
| --- | --- | --- | --- | --- |
| Cellular Component | cell part | 1:2:2 | 0.2:0.3:0.3 | Ml |
| Cellular Component | cell | 1:2:2 | 0.2:0.3:0.3 | Ml |
| Cellular Component | organelle | 1:2:2 | 0.2:0.3:0.3 | Ml |
| Cellular Component | membrane | 0:1:1 | 0.0:0.2:0.2 | Ml |
| Molecular Function | catalytic activity | 89:127:120 | 19.3:19.7:20.3 | 0.914 |
| Molecular Function | binding | 448:587:550 | 91.1:97.0:93.1 | 0.001 |
| Molecular Function | transcription regulator activity | 1:5:5 | 0.2:0.8:0.8 | Ml |
| Biological Process | response to stimulus | 1:3:3 | 0.2:0.5:0.5 | Ml |
| Biological Process | cellular process | 91:131:124 | 19.7:20.3:21.0 | 0.876 |
| Biological Process | metabolic process | 90:132:124 | 19.5:20.5:21.0 | 0.832 |
| Biological Process | biological regulation | 2:9:10 | 0.4:1.4:1.7 | Ml |
| Biological Process | regulation of biological process | 2:9:10 | 0.4:1.4:1.7 | Ml |
| Biological Process | signaling | 1:2:2 | 0.2:0.3:0.3 | Ml |

Note: ^1^GJ: XI-MH63: XI-ZS97; MI: Without P-value.
