# Supplementary material for: Conversion between 100-million-year-old duplicated genes contributes to rice subspecies divergence
Source: BMC Genomics. 2021 Jun 19;22:460. doi: 10.1186/s12864-021-07776-y (PMC8214281; doi:10.1186/s12864-021-07776-y)
Supplement: Supplementary file 18 — Additional file 18: Table S11. Comparison of expression differences between converted and nonconverted gene pairs. [file 12864_2021_7776_MOESM18_ESM.docx]

**Table S11** Comparison of expression differences between converted and nonconverted gene pairs.

| **Genome** | **Sample** | **Converted gene** | | | **Nonconverted gene** | | |
| --- | --- | --- | --- | --- | --- | --- | --- |
|  |  | **Expression** | **>Log2** | **Rate** | **Expression** | **>Log2** | **Rate** |
| GJ | panicle | 363 | 219 | 60.33% | 2407 | 1493 | 62.03% |
|  | seedling | 359 | 217 | 60.45% | 2387 | 1518 | 63.59% |
| XI-MH63 | leaf | 404 | 237 | 58.66% | 2339 | 1479 | 63.23% |
|  | panicle | 388 | 222 | 57.22% | 2247 | 1437 | 63.95% |
| XI-ZS97 | leaf | 313 | 189 | 60.38% | 1856 | 1304 | 70.26% |
|  | root | 409 | 252 | 61.61% | 2294 | 1456 | 63.41% |
