# Supplementary material for: Conversion between 100-million-year-old duplicated genes contributes to rice subspecies divergence
Source: BMC Genomics. 2021 Jun 19;22:460. doi: 10.1186/s12864-021-07776-y (PMC8214281; doi:10.1186/s12864-021-07776-y)
Supplement: Supplementary file 20 — Additional file 20: Table S13. Comparison of the mean FPKM difference between converted and nonconverted gene pairs. [file 12864_2021_7776_MOESM20_ESM.docx]

**Table S13** Comparison of mean FPKM difference between converted and nonconverted gene pairs.

| **Genome** | **Sample** | **Converted gene** | **Nonconverted gene** | **P-value** |
| --- | --- | --- | --- | --- |
| GJ | panicle | 20.76 | 26.03 | 0.010 |
|  | seedling | 16.45 | 20.98 | 0.026 |
| XI-MH63 | leaf | 18.56 | 26.97 | 0.002 |
|  | panicle | 17.60 | 27.54 | 8.1×10^-6^ |
| XI-ZS97 | leaf | 18.37 | 25.22 | 0.009 |
|  | root | 21.17 | 26.36 | 0.024 |
