# Supplementary material for: Conversion between 100-million-year-old duplicated genes contributes to rice subspecies divergence
Source: BMC Genomics. 2021 Jun 19;22:460. doi: 10.1186/s12864-021-07776-y (PMC8214281; doi:10.1186/s12864-021-07776-y)
Supplement: Supplementary file 21 — Additional file 21: Supplemental Text. Gene conversion and occurrence patterns. [file 12864_2021_7776_MOESM21_ESM.docx]

**Supplemental Text**

**Conversion between 100-million-year-old duplicated genes contributes to rice subspecies divergence**

Chendan Wei^1,†^, Zhenyi Wang^1,†^, Jianyu Wang^1,†^, Jia Teng^1^, Shaoqi Shen^1^, Qimeng Xiao^1^, Shoutong Bao^1^, Yishan Feng^1^, Yan Zhang^1^, Yuxian Li^1,2^, Sangrong Sun^1,2^, Yuanshuai Yue^1^, Chunyang Wu^1^, Yanli Wang^1^, Tianning Zhou^1^, Wenbo Xu^1^, Jigao Yu^2,3^, Li Wang^1,*^, Jinpeng Wang^1,2,3, *^

^†^These authors contributed equally to this work.

^*^Address correspondence to Jinpeng Wang (Email: [wangjinpeng@ibcas.ac.cn](mailto:wangjinpeng@ibcas.ac.cn)) and Li Wang (Email: [wlsh219@126.com](mailto:wlsh219@126.com)).

^1^School of Life Sciences, and Center for Genomics and Computational Biology, North China University of Science and Technology, Tangshan, Hebei 063000, China

^2^University of Chinese Academy of Sciences, Beijing 100049, China

^3^State Key Laboratory of Systematic and Evolutionary Botany, Institute of Botany, Chinese Academy of Science, Beijing 100093, China

**Gene conversion and occurrence patterns**

The 2879 pairs of paralogues were identified between GJ and XI-ZS97. After the divergence of GJ and XI-ZS97, 318 pairs of paralogues were converted in GJ. Among them, 12 pairs (0.42%) had underwent WCV-I (bootstrap > 80%). 131 pairs (4.55%) had underwent WCV-II, and these gene pairs were further support WCV-I. Moreover, 175 pairs (6.08%) had underwent PCV, with an average DNA fragment length of 73.65 bp where were converted, and the longest fragment is 917 bp (64.08%). And 10 pairs of paralogues were found to have undergone both PCV and WCV-II, which may be due to many paralogues that have further undergone repeated conversion at a more recent time after the WCV. For example, *Oj11g0138900.01* and *Oj12g0135500.00* may have repeatedly undergone WCV-II and PCV after the formation of GJ. And the *Ks* between that pair of paralogues in GJ was 0.19, which was smaller than their *Ks* with the corresponding orthologues *Zs11g0407.01* and *Zs12g0396.01* in XI-ZS97, which were 0.42 and 0.23, respectively; and the amino acid identity ratio between that pair of paralogues was 0.48, which was larger than the corresponding orthologues *Zs11g0407.01* (0.15) and *Zs12g0396.01* (0.19) in XI-ZS97. After the divergence of GJ and XI-ZS97, 403 pairs of paralogues were converted in XI-ZS97. Among them, 7 pairs (0.24%) had underwent WCV-I (bootstrap > 80%). 174 pairs (6.04%) had underwent WCV-II, and these gene pairs were further supported by WCV-II. 247 pairs (8.58%) had underwent PCV, with an average length of the converted DNA fragment was 68.94bp, and the longest fragment was 612bp (33.94%). And 15 pairs were also found to have undergone both PCV and WCV-II. Similar results were obtained in the comparison between between GJ and XI-MH63, with 2788 pairs of paralogues between them. And 307 pairs were converted in GJ, more pairs of paralogues (436) were converted in XI-MH63 (**Supplemental Table 2**).

By compared the two XI varieties of XI-MH63 and XI-ZS97, we found that gene conversion continued to occur after the formation of the varieties. In XI-ZS97, we found that 80 pairs of paralogues were converted. Among them, 1 pair had underwent WCV-I with bootstrap values of 0.89 and 0.99; and 2 pairs had underwent WCV-II. The 77 pairs had underwent PCV, with an average length of the conversion DNA fragment is 71.74 bp, and the longest is 421 bp (27.68%). In XI-MH63, we found the similar occurrence of gene conversion, with 79 pairs of paralogues had been converted. Among them, 1 pair had underwent WCV-I, with bootstrap values of 0.93 and 0.99; 1 pair had underwent WCV-II. The 77 pairs had underwent PCV, with the average length of the conversion DNA fragment was 88.35 bp, and the longest was 1097 bp (21.23%).

**Gene conversion in *Setaria***

In order to compare the pattern of gene conversion in other species, we selected *Setaria italica* and *Setaria viridis* to identify their homologous genes and infer possible gene conversion. 1,352 pairs of paralogues were identified in *Setaria italica*, 2,292 pairs of paralogues were identified in *Setaria viridis.* 29,658 pairs of orthologues were identified between the two species, and 1,637 quartets were finally obtained. After removed the highly divergent sequences, 1,494 quartets were identified. There was no WCV-I in either *Setaria italica* or *Setaria viridis*. There was one pairs of paralogues had underwent WCV-II in *Setaria italica*, while no WCV-II was inferred in *Setaria viridis*. Meanwhile, there were 47 pairs of paralogues had underwent PCV in *Setaria italica* and 84 pairs of paralogues had underwent PCV in *Setaria viridis*. Finally, the gene conversion rate is 0.033 and 0.056 in *Setaria italica* and in *Setaria viridis,* respectively.

We found that the gene conversion rate near the telomere in *Setaria italica* and in *Setaria viridis* did not appear to be significantly higher than that in other regions of the chromosomes (**Table S15; Fig. S10**). However, based on the division of rice chromosomal regions, we reanalyzed the gene conversion rate on the corresponding orthologous regions in *Setaria italica* and *Setaria viridis* genomes (**Table S16**). We also found that the similar phenomenon of higher terminal gene conversion rate was presented. We estimated a 1.3-fold increase in conversion rate within the subtelomeric region of 0-2 Mb to chromosome termini in *Setaria italica* (P-value = 2.00×10^-05^, permutation). In *Setaria viridis*, 1.2-fold increase in conversion rate within the subtelomeric region of 0-4 Mb to chromosome termini(P-value = 1.00×10^-04^, permutation).
