# Supplementary material for: Conversion between 100-million-year-old duplicated genes contributes to rice subspecies divergence
Source: BMC Genomics. 2021 Jun 19;22:460. doi: 10.1186/s12864-021-07776-y (PMC8214281; doi:10.1186/s12864-021-07776-y)
Supplement: Supplementary file 23 — Additional file 23: Fig. S9. Homologous dot plot between GJ and Setaria italica. The best, secondary, and other matched homologous gene pairs output by Blast were dotploted by red, blue, and gray colors in this figure. [file 12864_2021_7776_MOESM23_ESM.pdf]

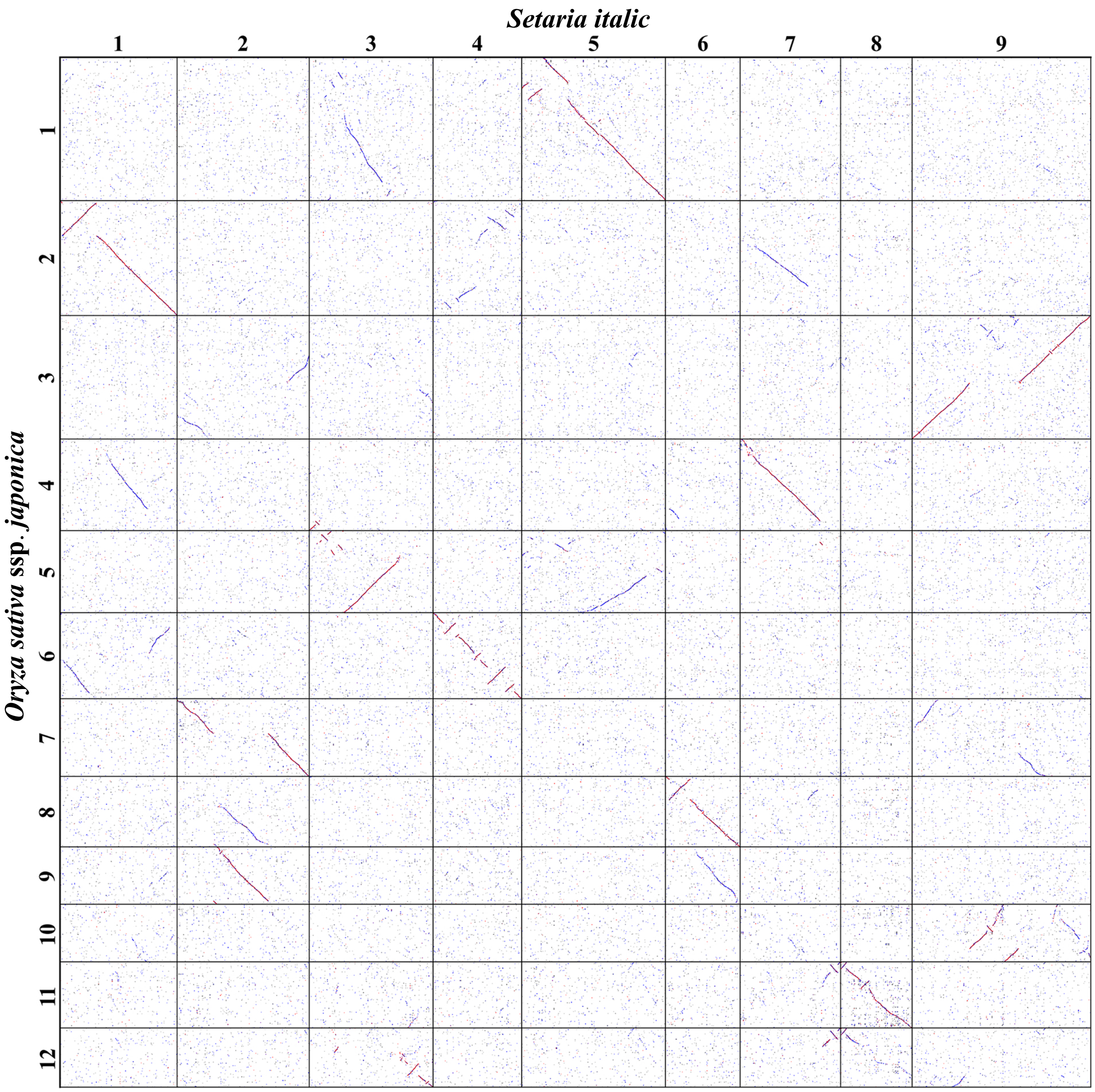
Setaria italic
1
2
3
4
5
6
7
8
9
Oryza sativa ssp. japonica
1
2
3
4
5
6
7
8
9
10
11
12
