# Supplementary material for: Conversion between 100-million-year-old duplicated genes contributes to rice subspecies divergence
Source: BMC Genomics. 2021 Jun 19;22:460. doi: 10.1186/s12864-021-07776-y (PMC8214281; doi:10.1186/s12864-021-07776-y)
Supplement: Supplementary file 24 — Additional file 24: Table S14. Distribution of paralogues and converted genes pairs in Setaria italica and Setaria viridis. [file 12864_2021_7776_MOESM24_ESM.docx]

**Table S14.** Distribution of paralogues and converted gene pairs in *Setaria italica* and *Setaria viridis*.

| **Paralogous chromosomes** | **Paralogous chromosomes of rice** | ***Setaria italica*** | | ***Setaria viridis*** | |
| --- | --- | --- | --- | --- | --- |
|  |  | **Paralogues** | **Converted pairs** | **Paralogues** | **Converted pairs** |
| chr01-chr04 | chr02-chr06 | 244 | 9 (3.69%) | 245 | 17 (6.94%) |
| chr01-chr07 | chr02-chr04 | 233 | 3 (1.29%) | 237 | 15 (6.33%) |
| chr02-chr06 | chr08-chr09 | 153 | 7 (4.58%) | 153 | 10 (6.54%) |
| chr02-chr09 | chr03-chr07 | 168 | 6 (3.57%) | 169 | 10 (5.92%) |
| chr03-chr05 | chr01-chr05 | 368 | 10 (2.72%) | 370 | 18 (4.86%) |
| chr03-chr09 | chr03-chr12 | 45 | 1 (2.22%) | 48 | 1 (2.08%) |
| chr03, 07-chr08 | chr11-chr12 | 153 | 5 (3.27%) | 155 | 4 (2.58%) |
| chr09-chr09 | chr03-chr10 | 110 | 7 (6.36%) | 117 | 9 (7.69%) |
| All |  | 1474 | 48 (3.26%) | 1494 | 84 (5.62%) |
