# Supplementary material for: Conversion between 100-million-year-old duplicated genes contributes to rice subspecies divergence
Source: BMC Genomics. 2021 Jun 19;22:460. doi: 10.1186/s12864-021-07776-y (PMC8214281; doi:10.1186/s12864-021-07776-y)
Supplement: Supplementary file 25 — Additional file 25: Table S15. Relationship between gene physical location and gene conversion in Setaria italica and Setaria viridis. [file 12864_2021_7776_MOESM25_ESM.docx]

**Table S15.** Relationship between gene physical location and gene conversion in *Setaria italica* and *Setaria viridis*.

| **Distance to telomere** | **<2 Mb** | **2-4 Mb** | | **4-6 Mb** | | **6-8 Mb** | | **8-10 Mb** | | **>10 Mb** | **All** |
| --- | --- | --- | --- | --- | --- | --- | --- | --- | --- | --- | --- |
| *Setaria italica* | | | | | | | | | | | |
| All converted | 17 | 13 | 9 | | 10 | | 11 | | 36 | | 96 |
| Paraloge genes | 446 | 399 | 261 | | 295 | | 284 | | 1159 | | 2844 |
| Mean converted rate | 3.80% | 3.25% | 3.38% | | 3.55% | | 3.73% | | 3.76% | | 3.38% |
| *Setaria viridis* | | | | | | | | | | | |
| All converted | 13 | 34 | 16 | | 12 | | 13 | | 77 | | 165 |
| Paraloge genes | 467 | 417 | 263 | | 306 | | 305 | | 1169 | | 2927 |
| Mean converted rate | 2.80% | 8.17% | 6.45% | | 4.18% | | 4.40% | | 6.16% | | 5.64% |
