# Supplementary material for: Conversion between 100-million-year-old duplicated genes contributes to rice subspecies divergence
Source: BMC Genomics. 2021 Jun 19;22:460. doi: 10.1186/s12864-021-07776-y (PMC8214281; doi:10.1186/s12864-021-07776-y)
Supplement: Supplementary file 27 — Additional file 27: Table S16. Relationship between gene physical location and gene conversion of Setaria italica and Setaria viridis in rice orthologous regions. [file 12864_2021_7776_MOESM27_ESM.docx]

**Table S16.** Relationship between gene physical location and gene conversion of *Setaria italica* and *Setaria viridis* in rice orthologous regions.

| **Distance to telomere** | **<2 Mb** | **2-4 Mb** | **4-6 Mb** | **6-8 Mb** | **8-10 Mb** | **>10 Mb** | **All** |
| --- | --- | --- | --- | --- | --- | --- | --- |
| *Setaria italica* | | | | | | | |
| All converted | 21 | 15 | 12 | 14 | 9 | 25 | 96 |
| Paraloge genes | 519 | 525 | 465 | 353 | 272 | 710 | 2844 |
| Mean converted rate | 4.04% | 2.84% | 2.46% | 3.86% | 3.25% | 3.48% | 3.38% |
| *Setaria viridis* | | | | | | | |
| All converted | 17 | 39 | 17 | 23 | 21 | 48 | 165 |
| Paraloge genes | 438 | 534 | 414 | 370 | 333 | 838 | 2927 |
| Mean converted rate | 7.01% | 7.25% | 5.01% | 5.51% | 4.78% | 4.36% | 5.64% |
